# Phantom-Free Geometric Refinement for Industrial CBCT Using Physical Constraints and a Normalized Low-Rank Projection Prior

**DOI:** 10.3390/s26165161

**Published:** 2026-08-14

**Authors:** Yanxu Sun, Xingyuan Bian, Igor A. Konyakhin, Junning Cui

**Affiliations:** 1 Center of Ultra-Precision Optoelectronic Instrument Engineering, Harbin Institute of Technology, Harbin 150080, China; 22b901030@stu.hit.edu.cn (Y.S.); bianxingyuan@163.com (X.B.); 2 Key Laboratory of Ultra-Precision Instruments and Intelligence of the Ministry of Industry and Information Technology, Harbin Institute of Technology, Harbin 150080, China; 3 Higher School of Engineering and Technology, ITMO University, Kronverksky Pr. 49, Bldg. A, St. Petersburg 197101, Russia; iakoniakhin@itmo.ru

**Keywords:** industrial cone-beam CT, phantom-free geometric refinement, projection reindexing, physical constraints, center-of-mass trajectory, normalized nuclear norm

## Abstract

**Highlights:**

**What are the main findings?**

- A periodic center-of-mass trajectory loss and physical bounds define a scan-specific search over bounded detector-offset and orientation corrections.
- A controlled industrial object benchmark, including a public epipolar-consistency baseline, quantitatively confirms the comparative image-quality advantage of the proposed refinement.

**What are the implications of the main findings?**

- The framework supports scan-specific geometric refinement when a dedicated calibration phantom cannot be deployed.
- Across 20 fixed-ROI slices, the proposed method achieved a mean SSIM of 0.9933 and an NRMSE of 0.0321, outperforming MI-PSO, PR, Open-ECC-R, and the uncalibrated reconstruction under the evaluated conditions.

**Abstract:**

Geometric misalignment degrades industrial cone-beam computed tomography (CBCT), particularly when a dedicated calibration phantom cannot be deployed during object acquisition. This study presents a three-stage, scan-specific geometric refinement framework that searches a bounded five-coordinate correction space around a nominal geometry. Coarse candidates are screened using the normalized residual between a geometry-corrected center-of-mass trajectory and its best-fitting low-order periodic model. Translation- and rotation-dominant coordinates are then refined within system-specific physical bounds, and an energy-normalized nuclear-norm score of corrected row-wise sinograms is used for local correlation refinement. The periodic and low-rank terms are treated as object-dependent surrogate objectives rather than as standalone guarantees of physical parameter identifiability. An exact-ASTRA implementation check using a Shepp–Logan volume verified the detector-plane reindexing convention: applying the injected correction reduced valid-mask projection discrepancy to 35.2%, 13.7%, and 8.66% of the uncorrected values for small, medium, and large perturbations, respectively, with round-trip resampling NRMSE of 0.022–0.023 and a mean valid fraction of 98.4%. Three industrial datasets acquired with horizontal gantry CT, temperature-stage in situ CT, and vertical micro-CT provided comparative reconstruction evidence. In addition, a controlled reduced-resolution industrial object reprojection benchmark was used for direct comparison with MI-PSO, PR, and a stability-regularized implementation of the public epipolar-consistency formulation (Open-ECC-R). Over 20 fixed-ROI axial slices, Open-ECC-R increased the mean SSIM from 0.6852 ± 0.0454 for the uncalibrated reconstruction to 0.8450 ± 0.0155 and reduced the NRMSE from 0.5180 ± 0.0526 to 0.1671 ± 0.0125. The proposed method achieved the highest mean SSIM of 0.9933 ± 0.0002 and the lowest NRMSE of 0.0321 ± 0.0009. For the Bluetooth earphone dataset, local sagittal and axial MTF_50_ estimates increased from 0.84 to 0.94 lp/mm and from 0.45 to 1.05 lp/mm, respectively. These results support scan-specific image-quality refinement around a nominal geometry while avoiding unsupported claims of absolute parameter recovery.

## 1. Introduction

Industrial cone-beam computed tomography (CBCT) is widely used for nondestructive testing, dimensional metrology, and internal-structure analysis [1,2,3]. Reconstruction fidelity is highly sensitive to the relative alignment of the X-ray source, rotation stage, and detector. Mechanical tolerances, gravity-induced deformation, and environmental thermal changes can perturb this geometry and introduce streaking, blurring, and spatial distortion, thereby reducing the reliability of defect analysis and dimensional measurements [4,5]. Geometric errors also interact with object-dependent effects. Metal-induced projection inconsistency, continuous-spectrum beam-hardening behavior, sinogram processing, and edge ringing can alter local edge-response metrics even when geometric alignment is improved [6,7,8]. Accordingly, geometric calibration results should be interpreted jointly with artifact-aware image-quality evidence rather than from a single sharpness metric.

Geometric calibration methods are commonly divided into phantom-based offline calibration and data-driven self-calibration. Phantom-based methods rely on precision reference objects and remain an established solution when a dedicated calibration scan is feasible [9,10]. For example, Yu et al. designed a low-rank texture phantom and used its global symmetry for geometric correction [11]. However, a static phantom-derived geometry may not represent the effective scanner state during restricted or temperature-dependent inspections, and deployment of a phantom can be impractical when the imaging volume is occupied by a large component or an in situ loading device.

Phantom-free or scan-specific calibration methods infer geometric information from the acquired projections. Image domain approaches optimize autofocus, entropy, mutual information, or related projection/reconstruction-consistency measures [12,13,14]. Feature- and registration-based approaches track structures across views or register measured projections to a reconstructed volume [15,16,17]. More recent studies have exploited data redundancy conditions for spiral CT [18], fully automatic fiducial localization for non-circular orbits [19], self-operating image similarity searches without a dedicated calibration phantom [20], or hybrid metrology and projection-consistency refinement for robotic CBCT [21]. These methods differ substantially in trajectory, prior-image or fiducial requirements, number of estimated parameters, and availability of implementations. MI-PSO and projection registration (PR) were selected as primary implemented baselines because they represent global image similarity optimization and projection/correspondence-based refinement. To provide the requested direct comparison with a projection redundancy method, the revised study additionally evaluates Open-ECC-R, a stability-regularized implementation of the publicly documented epipolar-consistency formulation of Aichert et al. [22], on the same controlled reduced-resolution industrial object projection dataset.

The central difficulty is, therefore, not merely the absence of a calibration phantom, but the combination of nonconvex optimization, partial coupling among geometric coordinates, and object-dependent projection information. A defensible scan-specific method should distinguish three different claims: implementation correctness of a candidate geometric transformation, improvement of reconstruction quality, and recovery of the underlying physical detector pose. The first two can be evaluated from projection and image evidence, whereas the third requires an independently identifiable objective or an external metrological reference.

To address these issues, this study proposes a three-stage phantom-free geometric refinement framework around a nominal circular CBCT geometry. Low-dimensional projection continuity is used only as qualitative motivation. A periodic center-of-mass trajectory (CMT) residual screens coarse candidates, physically constrained block-coordinate updates restrict the search to mechanically plausible corrections, and a normalized nuclear-norm sinogram score (NNSS) provides local projection correlation refinement. The output is interpreted as an effective scan-specific correction vector that improves the evaluated projection/reconstruction consistency, not as an independently traceable estimate of the complete detector pose.

The principal contributions are as follows. First, the study formulates a reproducible bounded correction search that does not require a dedicated phantom during object acquisition. Second, the revised analysis explicitly separates the roles and limitations of the two surrogate objectives: the fitted periodic CMT residual is used for coarse trajectory screening, whereas the energy-normalized NNSS is used only for local correlation refinement. Third, the candidate geometry is applied through a verified two-dimensional detector reindexing convention, and the implementation is checked using exact ASTRA forward projections and round-trip resampling. Fourth, the framework is evaluated on three industrial datasets, while claims of absolute five-parameter recovery, global optimality, and dimensional traceability are avoided. Fifth, direct quantitative comparison with MI-PSO, PR, and Open-ECC-R is reported using fixed-ROI SSIM, gradient-SSIM, NRMSE, and PSNR.

## 2. Materials and Methods

This section formulates phantom-free geometric refinement as a hierarchical bounded search around a nominal CBCT geometry. The geometric parameterization is first defined, followed by the projection sequence motivation, the approximate low-rank prior, the three-stage correction procedure, and an explicit discussion of objective identifiability and implementation verification.

### 2.1. Geometric Model of Cone-Beam CT

**Figure 1:**
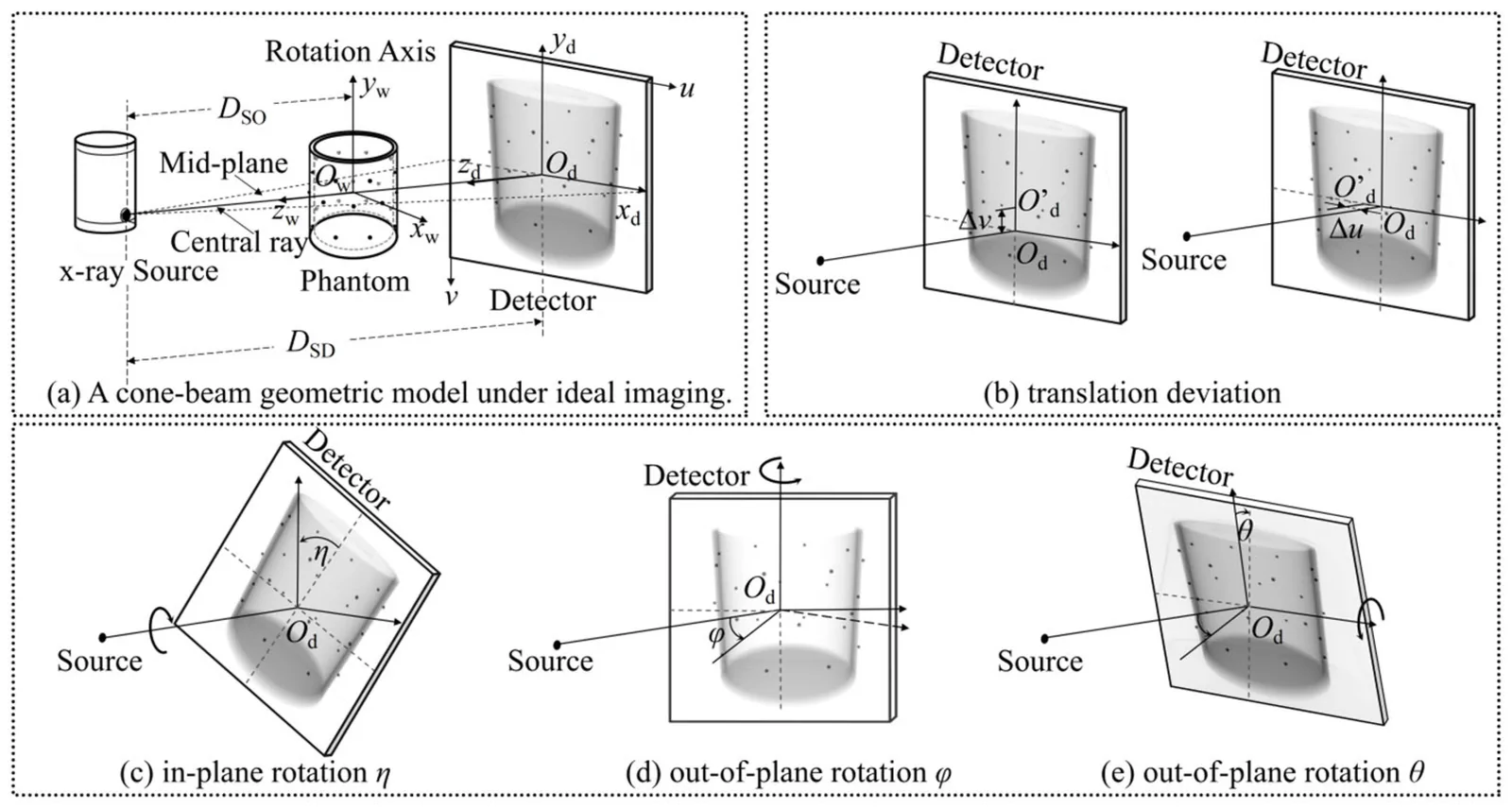
Circular CBCT geometry and definitions of the estimated detector-offset and detector orientation deviations.

A standard circular CBCT geometry is adopted, as illustrated in Figure 1. The complete nominal scanner geometry is described by the seven-parameter vector Θ_0_. In the present implementation, the source-to-object and source-to-detector distances are fixed at their nominal scanner values, whereas five correction coordinates—two detector offsets and three detector orientation angles—are optimized as bounded deviations around the nominal geometry. These coordinates define an effective detector reindexing rather than a complete, independently traceable physical error model. Fixing *D*_SO_ and *D*_SD_ avoids the unrestricted scale ambiguity that would arise if unknown object scale and absolute scanner magnification were estimated simultaneously from the same projection data:

(1) $$\Theta_{0}={\left[D_{SO,0},D_{SD,0},u_{0,0},v_{0,0},\eta_{0},\varphi_{0},\theta_{0}\right]}^{T},$$

(2) $$\Delta \xi ={\left[\Delta u_{0},\Delta v_{0},\Delta \eta ,\Delta \varphi ,\Delta \theta \right]}^{T},$$

(3) $$\Theta \left(\Delta \xi \right)=\Theta_{0}+{\left[0,0,\Delta u_{0},\Delta v_{0},\Delta \eta ,\Delta \varphi ,\Delta \theta \right]}^{T}.$$

Here, *D*_SO_ and *D*_SD_ denote the source-to-object and source-to-detector distances, respectively, (*u*_0_, *v*_0_) denotes the principal-point position on the detector, *η* is the in-plane detector rotation (roll), and *φ* and *θ* are the out-of-plane yaw and pitch angles, respectively.

For a three-dimensional point *P*(*x*, *y*, *z*) in the object, its projection *p*(*u*, *v*) on the detector at angle *β* is described by the perspective mapping H:

(4) $$p\left(u,v\right)=H\left(P,\beta ;\Theta \right).$$

In practical scans, mechanical misalignment causes the measured projection trajectory to deviate from the nominal mapping. The computational objective is, therefore, to identify an effective bounded correction vector using the acquired projections, nominal scanner geometry, and broad physical limits. Because the surrogate objectives used below are not independently traceable pose measurements, the resulting vector is interpreted as a scan-specific geometric correction rather than a complete physical calibration of the detector pose.

### 2.2. Theoretical Foundations

The proposed strategy is motivated by two complementary concepts: smooth angular evolution of projection descriptors and concentration of angular correlation after geometric correction. Neither concept is treated as an exact identifiability theorem, and neither objective is used as a substitute for independent metrological ground truth.

**Figure 2:**
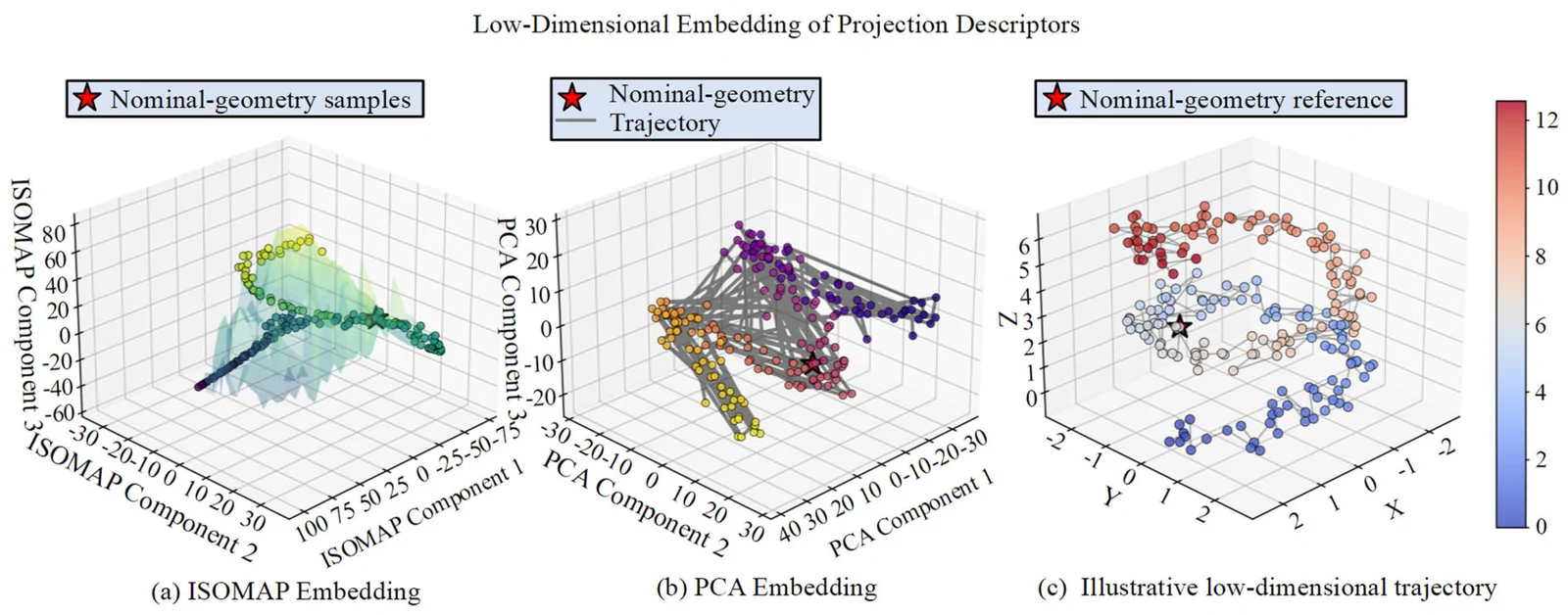
Low-dimensional visualization of simulated projection descriptors under geometric perturbations: (**a**) nonlinear ISOMAP embedding, (**b**) linear PCA embedding used as a reference, and (**c**) an illustrative trajectory in reduced coordinates. The embeddings motivate a structured search but are not optimization coordinates or evidence of exact manifold identifiability. Point color identifies the simulated perturbation condition indexed 0–12 on the color bar; red stars mark nominal-geometry reference samples, and the gray line in panel (**b**) shows the nominal-geometry trajectory.

Low-dimensional projection-sequence hypothesis: Projection images acquired over a continuous circular trajectory vary smoothly with projection angle, and suitable projection descriptors may, therefore, exhibit a low-dimensional continuous structure in a high-dimensional observation space. Figure 2a uses isometric feature mapping (ISOMAP) to visualize nonlinear neighborhood continuity under simulated geometric perturbations [23,24]. Figure 2b shows a principal component analysis (PCA) embedding as a linear reference, illustrating that a low-dimensional projection can be obtained without implying a unique nonlinear manifold. Figure 2c provides an illustrative trajectory view in reduced coordinates. These embeddings are qualitative visualizations only: they do not define the optimization variables, an explicit manifold distance, or a manifold regularizer. The actual search domain is the physically feasible parameter set Ω, defined independently from the nominal geometry and mechanical tolerance bounds.

Normalized low-rank prior of geometry-corrected projection data: Tomographic data-consistency relations imply angular redundancy among physically valid views, but they do not imply exact low rank for an arbitrary discretized CBCT dataset. In this study, the nuclear norm is used only as an empirical correlation score. Energy normalization prevents candidates with reduced signal energy or larger cropped regions from appearing artificially favorable. The score is object-, trajectory-, and preprocessing-dependent and is not a standalone proof that a candidate geometry is physically correct. Nuclear-norm minimization, singular-value thresholding, and robust low-rank decomposition provide related algorithmic context [25,26,27]; however, the present NNSS is only a normalized empirical score and does not perform matrix completion, singular-value shrinkage, or low-rank-plus-sparse decomposition.

### 2.3. Three-Stage Geometric Refinement Framework

**Figure 3:**
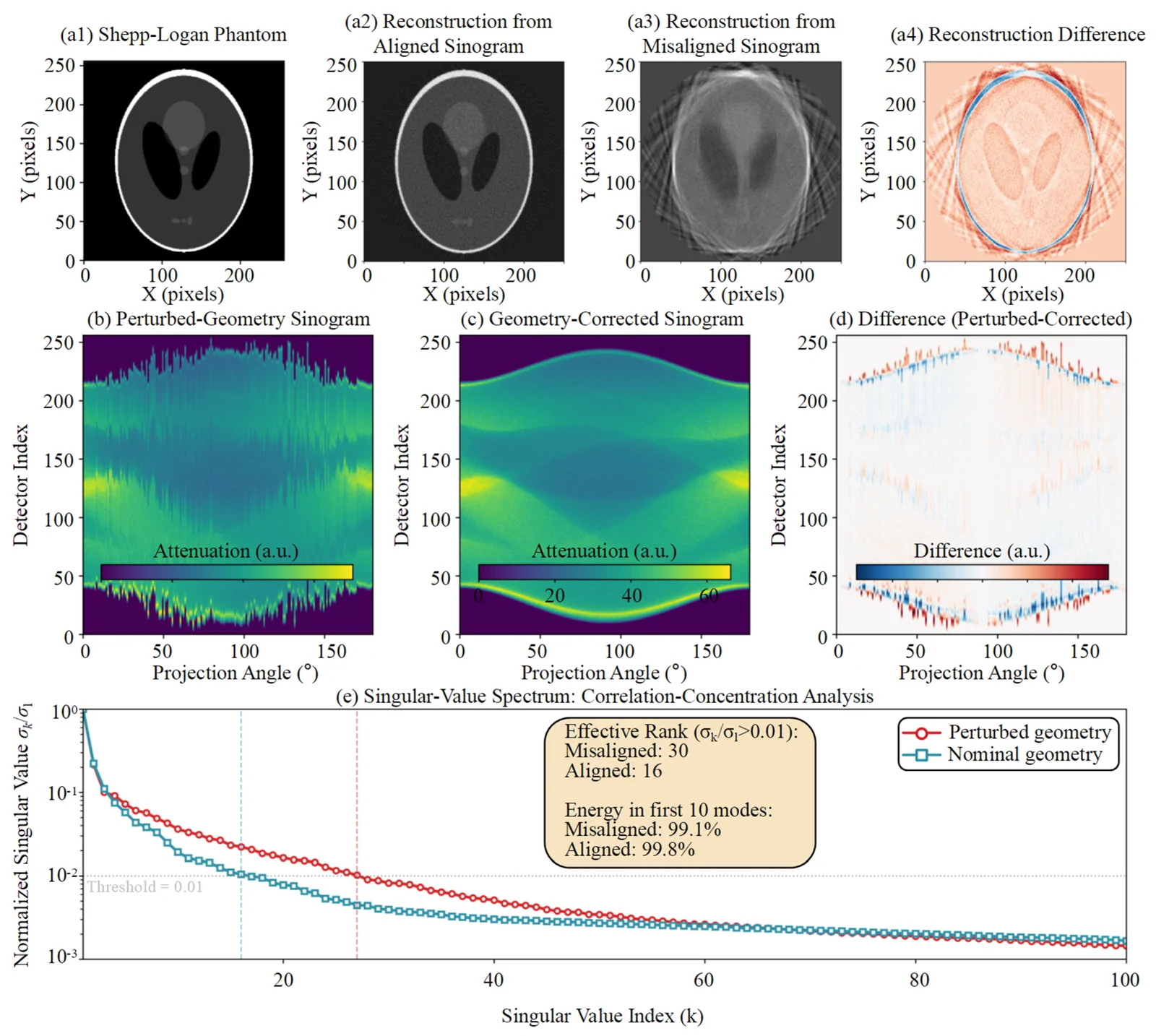
Empirical singular-value concentration of the central-row sinogram before and after geometric correction. The central row is shown for visualization, and the optimization uses the mean normalized nuclear-norm score over a fixed detector-row set. The threshold-based effective rank is only an illustrative descriptor for the evaluated simulation.

The proposed procedure uses coarse trajectory screening, physically constrained block-coordinate refinement, and approximate low-rank local fine-tuning to address nonconvexity, coordinate coupling, and feature dependency. The hierarchy is designed to provide a reproducible bounded correction rather than a global or uniquely identifiable pose estimate. The empirical singular-value concentration motivating the Stage III score is shown in Figure 3.

#### 2.3.1. Stage I: Coarse Periodic Trajectory Screening

Detector offsets and detector rotations can produce partially correlated changes in projection trajectories, creating multiple local minima in direct joint optimization. Stage I evaluates a discrete candidate set G covering feasible signs and coarse magnitudes. For each candidate, the projection sequence is geometrically reindexed, and its corrected center-of-mass trajectory C(Δξ) is extracted. The corrected trajectory is fitted by a two-harmonic periodic basis B. This stage is used only to reject clearly trajectory-inconsistent candidates and to provide a reproducible initialization—it is not treated as a complete five-coordinate identification step:

$$L_{CMT}\left(\Delta \xi \right)=\frac{min_{A}{\left\|C\left(\Delta \xi \right)\text{-}BA\right\|}_{F}^{2}}{{\left\|C\left(\Delta \xi \right)\right\|}_{F}^{2}+\varepsilon }$$

(5) $$\Delta \xi^{\left(0\right)}=arg\underset{\Delta \xi_{k}\in G}{min}L_{CMT}\left(\Delta \xi_{k}\right).$$

To compute ***C***(Δ***ξ***), let *I*(*u*, *v*, *β*; Δ***ξ***) denote the nonnegative attenuation image after dark-field correction, flat-field correction, logarithmic transformation, candidate geometric reindexing, view-wise background subtraction, and clipping of negative values. This definition prevents the air background from dominating the intensity-weighted trajectory.

The two-dimensional center of mass (*u*_cm_, *v*_cm_) for one projection view is calculated as:

(6) $$u_{cm}\left(\beta \right)=\frac{\sum_{u}\sum_{v}u\times I\left(u,v,\beta \right)}{\sum_{u}\sum_{v}I\left(u,v,\beta \right)},$$

(7) $$v_{cm}\left(\beta \right)=\frac{\sum_{u}\sum_{v}v\times I\left(u,v,\beta \right)}{\sum_{u}\sum_{v}I\left(u,v,\beta \right)}.$$

For candidate deviation Δ***ξ***, the trajectory matrix is formed by stacking the two center-of-mass coordinates over all *N* views:

(8) $$C\left(\Delta \xi \right)=\left[\begin{array}{c} c{\left(\beta_{1};\Delta \xi \right)}^{T}\\ M\\ c{\left(\beta_{N};\Delta \xi \right)}^{T} \end{array}\right],c\left(\beta_{1};\Delta \xi \right)=\left[\begin{array}{c} u_{cm}\left(\beta_{1};\Delta \xi \right)\\ v_{cm}\left(\beta_{1};\Delta \xi \right) \end{array}\right].$$

In Equation (5), A contains the least-squares coefficients of the fitted periodic trajectory, and ε is a small positive constant used only to prevent division by zero. The denominator makes the loss insensitive to the overall trajectory amplitude. Because the basis contains a constant term, a view-invariant centroid shift can be absorbed by the fitted coefficients; therefore, LCMT alone does not uniquely identify constant detector offsets. Likewise, low-order angular perturbations can be partially absorbed by the fitted sine/cosine coefficients. The loss is consequently used as a coarse trajectory consistency surrogate rather than as a physical parameter likelihood.

The candidate with the lowest periodic-fit residual is passed to Stage II. This step reduces the likelihood of an obviously unsuitable initialization, while the final correction still depends on the physical bounds, block updates, and local projection correlation criterion.

#### 2.3.2. Stage II: Physically Constrained Block-Coordinate Refinement


(9) $$\Delta \xi_{t}={\left[\Delta u_{0},\Delta v_{0}\right]}^{T},\Delta \xi_{r}={\left[\Delta \eta ,\Delta \varphi ,\Delta \theta \right]}^{T}.$$

The two blocks are updated alternately within the physically feasible parameter set:

(10) $$\Omega =\left\{\Delta \xi :l\leq \Delta \xi \leq u\right\}.$$

Here, l and u are the lower and upper bounds obtained from nominal scanner geometry and mechanical tolerances. The block-coordinate procedure reduces simultaneous coupling between detector offsets and detector rotations, and ***Ω*** is a box-constrained parameter set rather than a learned manifold:

(11) $$\Delta \xi_{t}^{k+1}=arg\underset{\Delta \xi_{t}\in \Omega_{t}}{min}L_{cons}\left(\Delta \xi_{t},\Delta \xi_{r}^{\left(k\right)}\right),$$

(12) $$\Delta \xi_{r}^{k+1}=arg\underset{\Delta \xi_{r}\in \Omega_{r}}{min}L_{cons}\left(\Delta \xi_{t}^{\left(k+1\right)},\Delta \xi_{r}\right).$$

L_cons_ in Equations (11) and (12) is the normalized periodic trajectory loss LCMT defined in Equation (5), evaluated after applying the candidate correction. Alternating translation- and rotation-dominant coordinate blocks within Ω reduces simultaneous coupling and excludes mechanically infeasible combinations. Although LCMT is invariant to an ideal view-invariant centroid translation, practical offset coordinates alter finite-support cropping and the complete two-dimensional detector reindexing before centroid extraction. The translation block is, therefore, interpreted as an empirical effective-correction search within the prescribed bounds, not as an independently identifiable detector position estimate. At a fixed search resolution, only strict objective decreases from a finite candidate set are accepted, so the implemented cycle terminates finitely—this does not imply a global optimum or coordinate-wise physical identifiability.

#### 2.3.3. Stage III: Normalized Low-Rank Local Fine-Tuning

After the block-coordinate update, remaining deviations are refined using angular correlation in the geometry-corrected projection data. The candidate transformation is first applied to the complete two-dimensional projection image. For each retained detector row r, Sr(Θ) denotes the corrected row-wise sinogram after the same object support cropping. Vertical translation, yaw, and pitch alter the two-dimensional resampling coordinates before row extraction, so the averaged score remains indirectly sensitive to vertical inconsistency. However, the row-wise construction does not explicitly model covariance between neighboring rows and is not claimed to identify all correction coordinates by itself:

(13) $${\tilde{S}}_{r}\left(\Theta \right)=\frac{S_{r}\left(\Theta \right)}{{\left\|S_{r}\left(\Theta \right)\right\|}_{F}+\varepsilon },$$

(14) $$L_{LR}\left(\Theta \right)=\frac{1}{\left|R\right|}\sum_{r\in R}\|{\tilde{S}}_{r}\left(\Theta \right){\|}_{*},$$

(15) $$\Theta^{*}=arg\underset{\Theta \in N\left(\Theta_{\mathrm{II}}\right)}{min}L_{LR}\left(\Theta \right)s.t.L_{CMT}\left(\Theta \right)\leq L_{CMT}\left(\Theta_{\mathrm{II}}\right).$$

Here, N(ΘII) is the Cartesian product of the final Stage II coordinate search intervals centered at the Stage II estimate. Stage III minimizes the NNSS, denoted LLR, only among candidates whose periodic trajectory loss does not exceed that of ΘII. The lexicographic constraint prevents the low-rank score from accepting an arbitrarily smoother or lower-energy dataset at the expense of a markedly poorer trajectory. The NNSS remains an empirical local selection criterion—no low-rank projection matrix is reconstructed, and no singular-value thresholding is applied to the measured data.

Local coordinate updates are accepted only when they strictly reduce LLR without increasing LCMT. At each fixed resolution, the candidate set is finite, so a complete coordinate cycle with no accepted update provides a deterministic stopping condition. The resulting vector is an effective bounded correction selected by the hierarchy. It should be assessed using projection/reconstruction evidence and should not be interpreted as a traceable five-parameter pose estimate.

#### 2.3.4. Identifiability Scope of the Surrogate Objectives

Let PB denote the orthogonal projector onto the columns of the periodic basis B, because B includes the constant vector, (I − PB)1 = 0. Thus, for an ideal view-invariant centroid shift d, the residual of C + 1dT is identical to the residual of C. This establishes that the fitted periodic residual cannot independently identify constant detector translations. The one-dimensional diagnostic profiles in Supplementary Figure S2 similarly show that LCMT and NNSS do not attain their minima at the injected value for every coordinate. Accordingly, the method is positioned as a bounded scan-specific refinement method. Independent physical parameter recovery would require additional redundancy relations, object features, prior-volume registration, or a metrological reference.

The complete procedure is summarized in Algorithm 1.
**Algorithm 1.** Physically constrained geometric refinement with normalized low-rank local fine-tuning**Step****Operation****Input**Measured projections *P = {P_n_}_n=1_^N^*; nominal geometry *Θ_0_*; physical bounds *Ω = [ℓ, u]* for *Δξ = [Δu_0_, Δv_0_, Δη, Δφ, Δθ]^T^*; coarse candidate set *G*; two-harmonic basis *B*; retained row set *ℛ*; normalization constant *ε*; Stage-II/III step vectors and stopping tolerances.**Output**Selected corrected geometry *Θ^⋆^ = Θ(Δξ^⋆^)* and corrected projections *$P^{⋆}$*, where *Θ(Δξ)* applies the bounded correction to *Θ_0_*.1Preprocess every *P_n_* by dark-/flat-field correction, logarithmic transformation, view-wise background subtraction, and clipping of negative values.**Stage I****Coarse periodic-trajectory screening**2**for all***Δξ_g_ ∈ G*  **do**3
 *Θ_g_*
*← Θ*
*(*
*Δξ*
*_g_*
*);*
*$P_{g}$ ←*
*Reindex(*
*P*
*, Θ_g_)*
4 Extract the corrected center-of-mass trajectory *C_g_ = C(Δξ_g_)* from the selected views.5
 *A_g_ ← arg min_A_ ‖C_g_ − BA‖_F_^2^*
6
 *L_CMT_(Δξ_g_) ← ‖C_g_ − BA_g_‖_F_^2^/(‖C_g_‖_F_^2^ + ε)*
7**end for**8*Δξ^(0)^ ← arg min_{Δξ_g_ ∈ G} L_CMT_(Δξ_g_)* ▷ Reproducible initialization only**Stage II****Physically constrained block-coordinate refinement**9Partition *Δξ_t_ = [Δu_0_, Δv_0_]^T^* and *Δξ_r_ = [Δη, Δφ, Δθ]^T^*.10Initialize *Δξ^II^ ← Δξ^(0)^* and *s ← s_II_^0^*.11**while***max_q_(s_q_/s_II,q_^min^) ≥ 1* and the maximum number of block cycles is not reached **do**12 updated *←*
**false**13 Construct a finite translation-block neighborhood *$N_{t}$(Δξ^II^; s) ⊂ Ω*.14
 *${\hat{\xi }}_{t}$ ←*
*arg min_{*
*ζ ∈ $N_{t}$*
*} L_CMT_(ζ, Δξ_r_^II^)*
15
 **if**
*L_CMT_(${\hat{\xi }}_{t}$*
*, Δξ_r_^II^) < L_CMT_(Δξ^II^) *
**then**
16  Update *Δξ_t_^II^ ← ${\hat{\xi }}_{t}$*; updated *←*
**true**.17
 **end if**
18 Construct a finite rotation-block neighborhood *$N_{r}$(Δξ^II^; s) ⊂ Ω*.19
 *${\hat{\xi }}_{r}$ ←*
*arg min_{*
*ζ ∈ $N_{r}$*
*} L_CMT_(Δξ_t_^II^, ζ)*
20
 **if**
*L_CMT_(Δξ_t_^II^, ${\hat{\xi }}_{r}$*
*) < L_CMT_(Δξ^II^) *
**then**
21  Update *Δξ_r_^II^ ← ${\hat{\xi }}_{r}$*; updated *←*
**true**.22
 **end if**
23 **if** updated = **false then**24  *s ← s / 2* ▷ Move to a finer fixed search resolution25
 **end if**
26**end while****Stage III****Normalized low-rank local fine-tuning**27*Θ_II_ ← Θ(Δξ^II^), Δξ^⋆^ ← Δξ^II^*, and *s ← s_III_^0^*.28Apply candidate reindexing to the complete two-dimensional projections.29**for all***r ∈ ℛ* **do**30 Form the corrected row-wise sinogram *S_r_(Θ)* using the fixed object-support crop.31
 *${\tilde{S}}_{r}$(Θ)* *←* *S_r_(Θ)/(‖S_r_(Θ)‖_F_ + ε)*
32**end for**33Define *L_LR_(Θ) = |ℛ|^−1^ ∑_r∈ℛ_ ‖${\tilde{S}}_{r}$(Θ)‖_∗_*.34**while** *max_q_(s_q_/s_III,q_^min^) ≥ 1* and the maximum number of local cycles is not reached **do**35 updated *←*
**false**36 **for**
*q = 1* to *5*  **do**37  Construct the finite coordinate neighborhood *$N_{q}$(Δξ^⋆^; s_q_) ⊂ N(Θ_II_) ∩ Ω*.38  *ℰ_q_ ← {Θ ∈ $N_{q}$: L_CMT_(Θ) ≤ L_CMT_(Θ_II_) + τ_CMT_}*.39  **if**
*ℰ_q_ ≠ ∅* **then**40
   *$\hat{\Theta }$ ← *
*arg min_{*
*Θ ∈ ℰ*
*_q_*
*} L_LR_(Θ)*
41
   **if** *L_LR_($\hat{\Theta }$) < L_LR_(Θ(Δξ*
*^⋆^*
*)) − τ_LR_*
**then**
42    Set *Δξ^⋆^* to the correction vector associated with $\hat{\Theta }$; updated *←*
**true**.43
   **end if**
44
  **end if**
45
 **end for**
46 **if** updated = **false then**47
  *s*
* ← *
*s/2*
48
 **end if**
49**end while**50*Θ^⋆^ ← Θ(Δξ^⋆^); $P^{⋆}$ ← Reindex(P, Θ^⋆^)*.51**return** *Θ^⋆^,$P^{⋆}$*

### 2.4. Implementation Details and Parameter Selection

The search domain is defined around the nominal geometry. For the industrial refinements, scanner-specific bounds did not exceed ±5 mm for detector offsets or ±3° for detector orientation coordinates. The reviewer-requested reduced-resolution diagnostic used a fixed two-harmonic model, ε = 10^−6^, 72 uniformly sampled fine views, and 36 coarse views. Stage I evaluated the zero candidate, four signed single-coordinate candidates at each of 0.35 and 0.75 of the bounds, and 48 seeded random candidates. Stage II used initial coordinate steps [0.50, 0.50, 0.30, 0.30, 0.30] (mm, mm, degrees, degrees, degrees), minimum steps [0.025, 0.025, 0.015, 0.015, 0.015], and at most eight block cycles. Stage III used initial steps [0.15, 0.15, 0.08, 0.08, 0.08], minimum steps [0.0125, 0.0125, 0.0075, 0.0075, 0.0075], and at most six cycles. Detector rows whose mean absolute projection energy exceeded 5% of the maximum row energy were retained with unit row stride. A Stage III candidate was eligible when LCMT did not exceed the Stage II value by more than 10^−5^ and was accepted only when NNSS decreased by more than 10^−8^. These settings were fixed before image-quality evaluation.

Stage III uses the same object support crop, detector-row set, and preprocessing for every candidate. Each row-wise sinogram is normalized before its nuclear norm is computed, and a local candidate is accepted only when LLR decreases and LCMT does not increase. Candidate generation and stopping rules are fixed before image-quality evaluation. No image domain regularization or singular-value thresholding is used during the geometric search; therefore, changes in reconstructed edge behavior cannot be attributed to an explicit NNSS-based image-smoothing operation.

#### 2.4.1. Convergence and Computational Complexity

Let |G| denote the number of Stage I candidates, Ns the number of sampled views, P the number of processed detector pixels per view, R the number of retained detector rows, U the cropped detector width, and N the number of Stage III views. Candidate-wise reindexing and CMT extraction scale approximately as O(|G|NsP) in Stage I and O(KIINP) in Stage II. The dominant Stage III cost comprises repeated two-dimensional resampling and row-wise singular-value decompositions, with approximate cost O(KIII[R·min(UN^2^,NU^2^) + NP]). The implementation was benchmarked using a custom computational workflow with ASTRA Toolbox v2.0.1 (CWI, Amsterdam, The Netherlands, and the University of Antwerp, Antwerp, Belgium) on an Intel Core i9-12900KF CPU (Intel Corporation, Santa Clara, CA, USA), 128 GB DDR5 RAM, and an NVIDIA GeForce RTX 3090 Ti GPU (NVIDIA Corporation, Santa Clara, CA, USA). Across 60 downsampled diagnostic runs using 36 views at 64 × 64 for coarse processing and 72 views at 96 × 96 for fine processing, Stage I, Stage II, and Stage III required 2.49 ± 0.12 s, 2.89 ± 0.12 s, and 5.67 ± 2.32 s, respectively, and total runtime was 11.06 ± 2.33 s, with 204.7 ± 7.2 CMT evaluations and 22.3 ± 13.0 NNSS evaluations. These timings characterize the implementation rather than the full-resolution reconstruction workflow.

For a representative standardized benchmark on the same computer, the proposed implementation required 10.31 s for 229 objective evaluations, compared with 6.51 s for the implemented MI-PSO benchmark (180 evaluations) and 2.88 s for the implemented projection registration benchmark (71 evaluations). The Open-ECC-R comparison required 51.79 s for 264 epipolar-consistency evaluations on 72 projections reduced to 96 × 96 pixels. These timings indicate the implementation scale—because the objectives and evaluation counts differ, they are not interpreted as accuracy-equivalent complexity measurements.

#### 2.4.2. Sensitivity, Model Mismatch, and Statistical Evaluation

The reviewer-requested diagnostics were performed in two tiers. First, exact ASTRA cone-vector forward projections were used to verify the detector axis convention, sign convention, and inverse reindexing operation for small, medium, and large injected perturbations. Second, one-dimensional profiles were evaluated with all other coordinates fixed at their injected values. The exact-ASTRA check confirmed that the geometric transformation itself is correctly implemented, whereas the objective profiles showed that the CMT and NNSS surrogates are not individually identifiable in every coordinate direction. This result motivated the revised scope and the removal of the earlier absolute parameter recovery claims. Additional stress tests varied harmonic order, ε, and physical-bound width. Varying ε from 10^−8^ to 10^−4^ changed the NNSS only in the sixth decimal place, while harmonic order and bound width changed the selected local solution, confirming that these settings must be fixed a priori and are not universal. Distance errors, severe view reduction, limited-angle acquisition, and time-varying geometry remain outside the validated scope.

Supplementary Table S1 and Supplementary Figure S1 summarize the exact-ASTRA reindexing verification, Supplementary Figure S2 and Table S2 summarize the one-dimensional objective profiles, and Supplementary Table S3 reports the computational benchmark. Component, parameter setting, initialization, and *D*_SO_/*D*_SD_ mismatch diagnostics are reported in Supplementary Tables S4–S7. Because the diagnostic profiles did not support independently identifiable five-coordinate recovery, Monte Carlo pose-error statistics are not presented as evidence of absolute calibration accuracy. Instead, the revised numerical study evaluates transformation fidelity, objective behavior, and computational reproducibility, while explicitly limiting the industrial results to comparative image-quality evidence.

#### 2.4.3. Public Epipolar-Consistency Baseline and Quantitative Evaluation

Open-ECC-R was implemented from the epipolar-consistency formulation reported by Aichert et al. [22]. The consistency metric operates on pairs of cone-beam projections through redundant epipolar-plane integrals and does not require a calibration phantom, a prior three-dimensional volume, or training data during optimization. The implementation used 72 uniformly sampled projections reduced from 1024 × 1024 to 96 × 96 pixels, 168 view pairs, the same nominal five-coordinate geometry representation used by the proposed method, and three seeded particle-swarm searches. A weak nominal-centered penalty and a boundary/stability check were used to avoid accepting an unstable edge-of-domain solution, and the nominal geometry was retained when no reproducible non-boundary minimum was obtained.

Because raw scanner projections were unavailable for this additional benchmark, the comparison used a controlled projection dataset generated by reprojecting an industrial Bluetooth earphone volume with the nominal cone-beam geometry. This dataset is, therefore, described as a controlled industrial object reprojection benchmark rather than as a raw acquisition calibration experiment. Open-ECC-R, MI-PSO, PR, and the proposed method used identical angular sampling, reduced detector resolution, reconstruction dimensions, slice coordinates, and evaluation region. The phantom-calibrated reconstruction was used only as a post hoc image-quality reference and was not supplied to any phantom-free optimization procedure.

Quantitative evaluation used 20 consecutive axial slices (447–466) and a fixed region of interest [x, y, width, height] = [348, 530, 292, 204] pixels containing the earphone structure. Each reconstructed volume was normalized once using its 0.5th and 99.5th percentiles over the complete fixed-ROI slab. No per-slice intensity fitting, histogram matching, or post-reconstruction spatial registration was applied. Mean SSIM, gradient-SSIM, normalized ROI NRMSE, PSNR, and their across-slice standard deviations were then calculated.

### 2.5. Experimental Setup and Evaluation Design

The framework was evaluated using a Shepp–Logan numerical implementation check, and three industrial datasets acquired with horizontal gantry CT, temperature-stage in situ CT, and vertical micro-CT. MI-PSO [13,28] and PR [29] were selected as complementary image similarity and correspondence-based baselines. Open-ECC-R [22] was added as a publicly documented projection-consistency baseline. The numerical study verifies the geometric transformation, examines objective profiles, and reports computational behavior—it is not presented as traceable recovery of the complete five-coordinate physical pose. The industrial datasets provide comparative reconstruction evidence, while the controlled industrial object reprojection benchmark provides fixed-ROI SSIM, gradient-SSIM, NRMSE, and PSNR comparisons against the phantom-calibrated post hoc reference.

**Figure 4:**
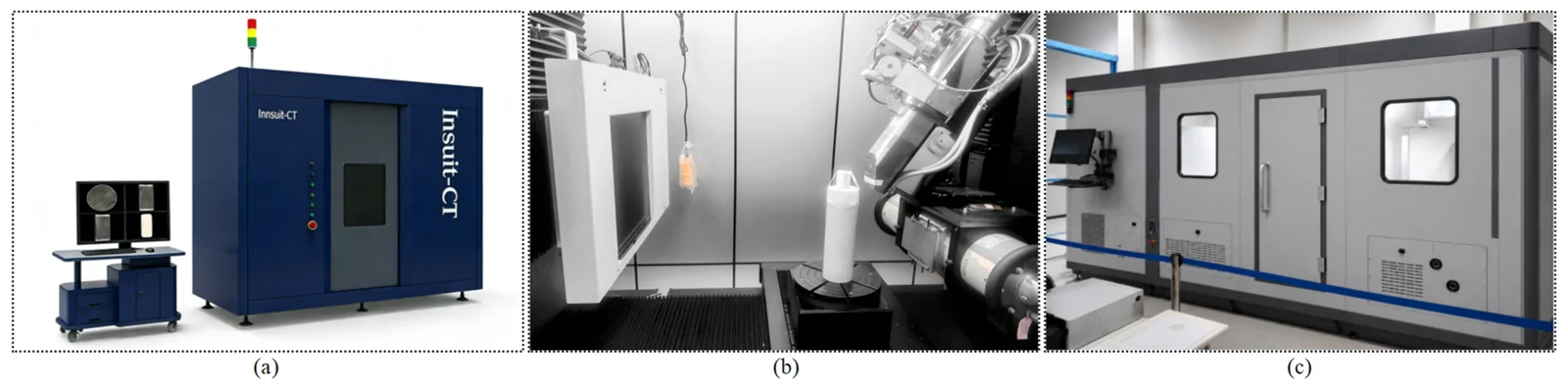
Experimental platforms: (**a**) Sanying nanoVoxel-4000 in situ CT, (**b**) Comet Yxlon FF35 vertical micro-CT, and (**c**) custom horizontal gantry CT for a high-density tungsten particle assembly.

Three X-ray CT platforms were used (Figure 4): a Comet Yxlon FF35 vertical micro-CT system (Comet Technologies Germany GmbH, Hamburg, Germany) for microelectronic inspection, a Sanying nanoVoxel-4000 system (Sanying Precision Instruments Co., Ltd., Tianjin, China) for temperature-stage in situ imaging of a heated rock core, and a custom horizontal gantry CT system developed at Harbin Institute of Technology (Harbin, China) for a high-density tungsten particle assembly. Acquisition settings were selected according to the attenuation and spatial resolution requirements of each object. The datasets, scanner configurations, and evaluation evidence are summarized in Table 1.

## 3. Results

### 3.1. Numerical Implementation Verification and Objective Profile Analysis

A 1024^3^ Shepp–Logan volume was downsampled to 128^3^ voxels for an exact ASTRA cone-vector diagnostic with 72 views and a deliberately non-square 96 × 128 detector. The nominal geometry used *D*_SO_ = 800 mm, *D*_SD_ = 1200 mm, and detector pitches of 2.54558 and 3.39411 mm along the two detector axes. The non-square detector made the ASTRA output ordering and the physical detector u/v axes distinguishable. Small, medium, and large coupled corrections were injected, and the inverse correction was applied through the same two-dimensional reindexing operation used by the refinement framework.

The numerical check isolates implementation fidelity rather than estimator accuracy. Projection discrepancy was evaluated only over pixels valid under both the forward perturbation and the inverse interpolation mask. The corrected-to-uncorrected valid-mask NRMSE ratios were 0.3523, 0.1365, and 0.0866 for the small, medium, and large cases, corresponding to discrepancy reductions of 64.8%, 86.3%, and 91.3%. Round-trip resampling NRMSE remained between 0.0222 and 0.0233, and the mean valid-mask fraction was 98.36%. These results verify the detector axis convention and the implemented inverse reindexing operation.

Because the full-field differences are concentrated near object and detector boundaries and are difficult to discern at manuscript scale, the numerical verification is summarized in Table 2 rather than by nearly identical full-projection panels. Supplementary Figure S1 shows the valid-mask NRMSE reduction and round-trip resampling error for the three perturbation levels.

One-dimensional objective profiles were then evaluated with all other coordinates fixed at their injected values. The projection domain NRMSE formed a minimum near the injected coordinate after the detector convention was corrected, whereas LCMT and NNSS did not provide a truth-aligned minimum for every coordinate. Across 15 coordinate profiles, the CMT minimum coincided with the injected value in 0 cases and the NNSS minimum in 4 cases. This finding is consistent with the invariance of the fitted periodic residual to ideal constant centroid shifts and supports the revised interpretation of LCMT and NNSS as bounded screening/refinement surrogates rather than standalone parameter identification criteria.

Section 3.2, Section 3.3 and Section 3.4, therefore, assess comparative reconstruction quality in the presence of real noise, beam hardening, metal artifacts, and temperature-dependent scanner changes. Because independent geometric ground truth is unavailable for these datasets, the industrial results are interpreted as relative image-quality evidence rather than absolute pose or dimensional accuracy.

#### Objective Sensitivity, Runtime, and Scope of Statistical Claims

The exact-ASTRA verification is summarized in Supplementary Figure S1 and Table S1, whereas the one-dimensional objective profiles and their minimum-count summary are provided in Supplementary Figure S2 and Table S2. They show that the detector transformation is numerically consistent, but that the two surrogate objectives are not independently identifiable across all five coordinate directions. The revision, therefore, removes the previously reported sub-pixel and sub-0.02° pose-recovery claims and does not present Monte Carlo pose-error statistics that the objective cannot support. Component diagnostics separate the roles of screening, bounded block refinement, and NNSS local selection (Table S4), and harmonic order, ε, and bound-width effects are summarized in Table S5. Ten initialization trials produced similar final surrogate values but different correction vectors (Table S6), confirming that objective convergence is not equivalent to a unique physical solution. A ±1% *D*_SO_/*D*_SD_ mismatch diagnostic altered the surrogate values and selected local correction (Table S7), supporting the decision to keep the distance parameters fixed and to state this model dependence explicitly.

This diagnostic outcome defines the applicability of the method and prevents overinterpretation of the industrial reconstructions. Initialization dependence, distance mismatch, sparse views, limited-angle data, and intra-scan drift are consequently treated as operating limitations rather than as validated absolute-calibration capabilities. The numerical evidence supports bounded image-quality refinement and implementation fidelity, not coordinate-wise metrological recovery.

### 3.2. Real-World Application I: Horizontal Gantry CT of a Tungsten Particle Assembly

**Figure 5:**
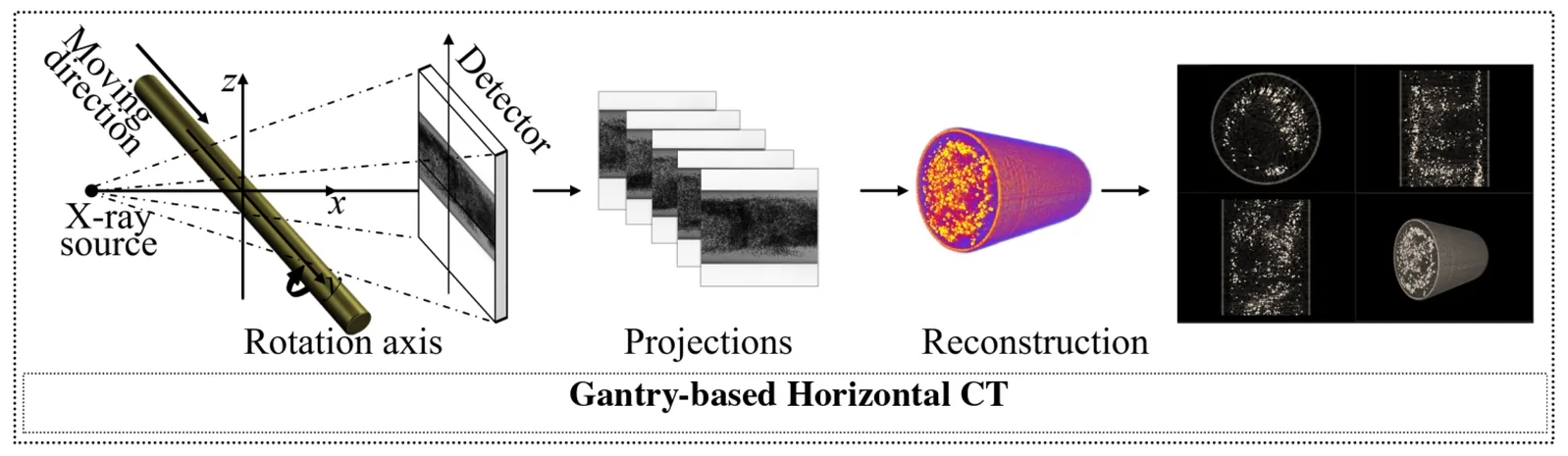
Horizontal gantry CT of a cylindrical high-density tungsten particle assembly and the corresponding reconstruction workflow.

**Figure 6:**
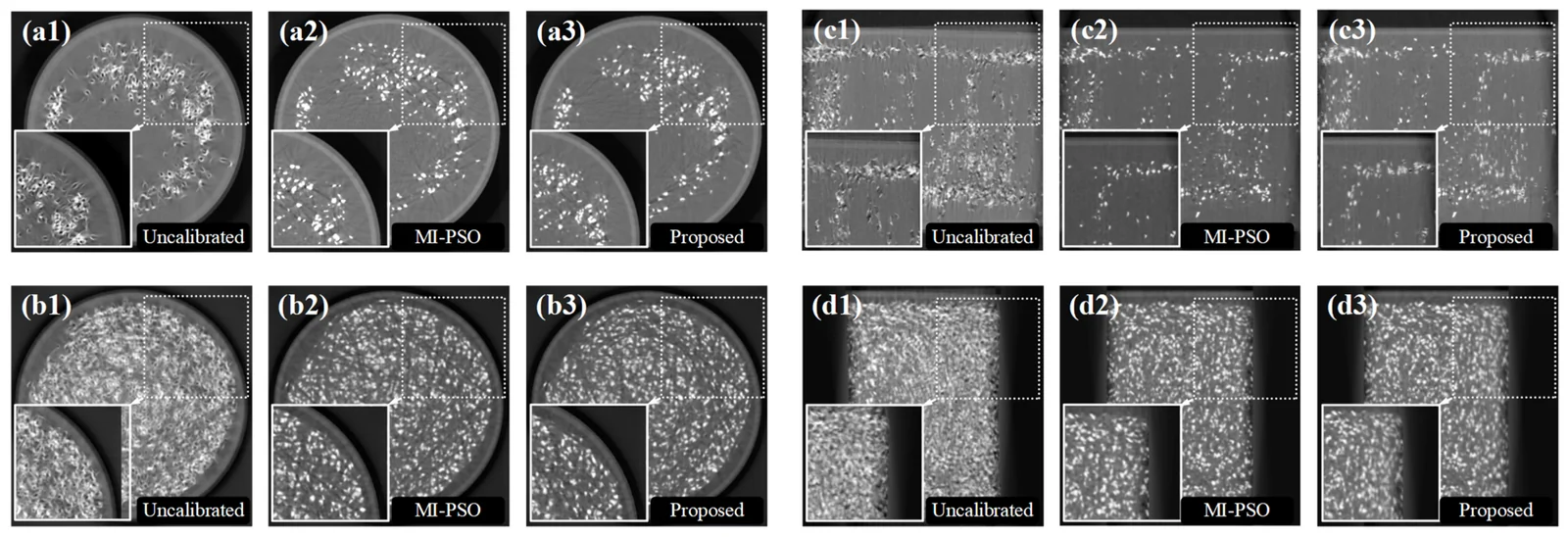
Reconstructed slices of the cylindrical tungsten particle assembly: (**a1**) first transverse slice, uncalibrated; (**a2**) first transverse slice, MI-PSO; (**a3**) first transverse slice, proposed framework; (**b1**) second transverse slice, uncalibrated; (**b2**) second transverse slice, MI-PSO; (**b3**) second transverse slice, proposed framework; (**c1**) first longitudinal slice, uncalibrated; (**c2**) first longitudinal slice, MI-PSO; (**c3**) first longitudinal slice, proposed framework; (**d1**) second longitudinal slice, uncalibrated; (**d2**) second longitudinal slice, MI-PSO; and (**d3**) second longitudinal slice, proposed framework. Insets enlarge the indicated regions.

A cylindrical tungsten particle assembly was scanned using a custom horizontal gantry CT system (Figure 5). The long mechanical support is susceptible to gravity-dependent deformation, while the smooth cylindrical exterior provides limited distinct projection features. Figure 6 compares the proposed method with the evaluated MI-PSO baseline.

The MI-PSO reconstruction retained radial streaks and locally blurred interfaces around several high-density particles in the displayed slices (Figure 6). The proposed bounded correction produced clearer particle boundaries and reduced view-inconsistent streaking in the highlighted regions. Because no independent geometric ground truth was available for this scan, these differences are interpreted as comparative image-quality evidence rather than as coordinate-wise calibration accuracy.

### 3.3. Real-World Application II: Temperature-Stage In Situ CT of a Heated Rock Core

**Figure 7:**
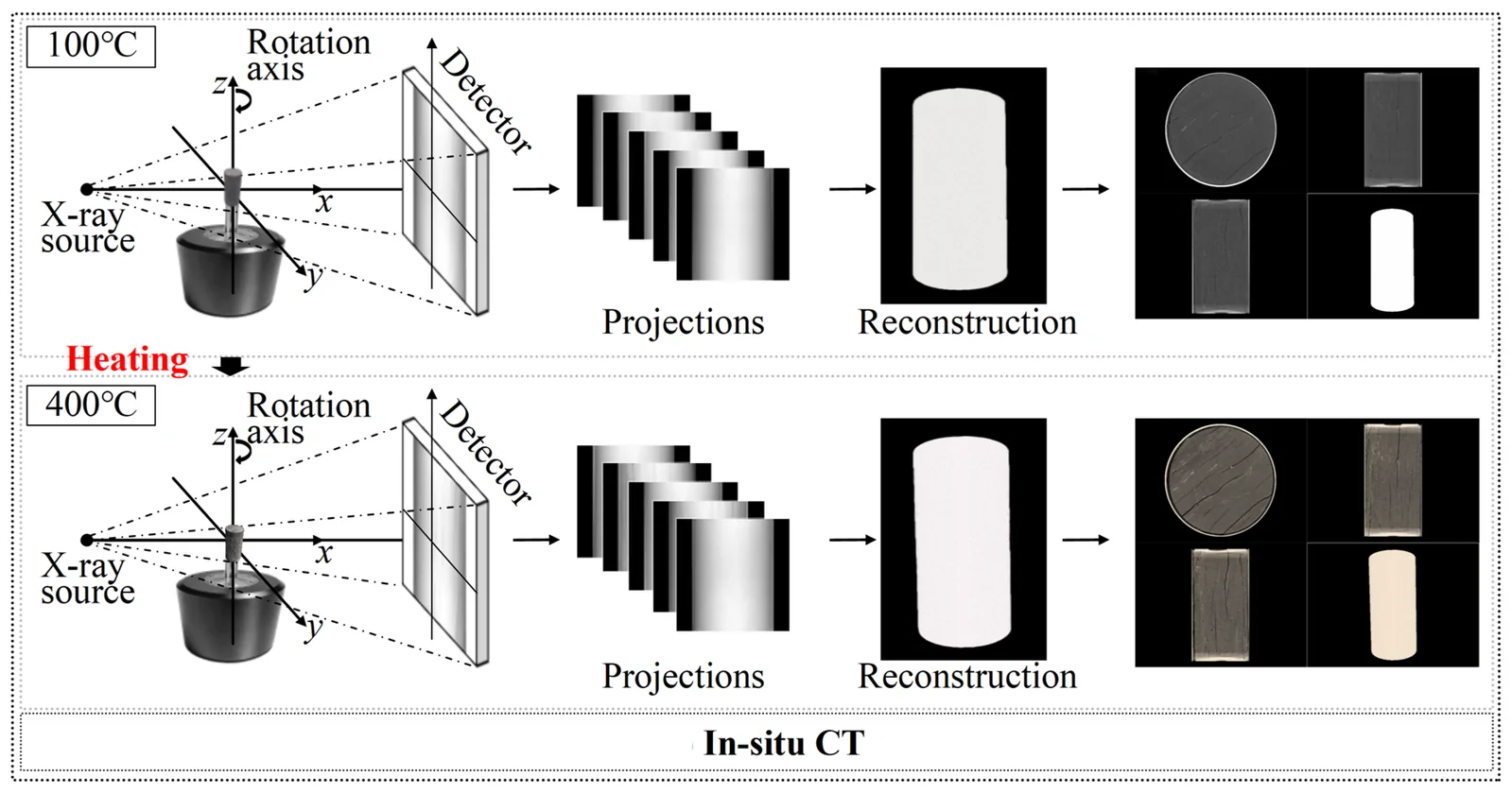
In situ CT acquisition of a shale rock core at temperature stages between 100 and 400 °C. Thermal loading may produce temperature-dependent changes in the effective scanner geometry.

A shale rock core was imaged at selected temperature stages between 100 and 400 °C to assess scan-specific phantom-free geometric refinement under temperature-dependent scanner changes (Figure 7). Thermal loading can change the effective rotation-center position and scanner geometry both between temperature stages and, if equilibrium is not fully reached, during an individual acquisition. The present implementation estimates one best static equivalent correction for each projection dataset. It, therefore, compensates the dominant scan-averaged inconsistency but does not recover a continuously varying intra-scan trajectory.

**Figure 8:**
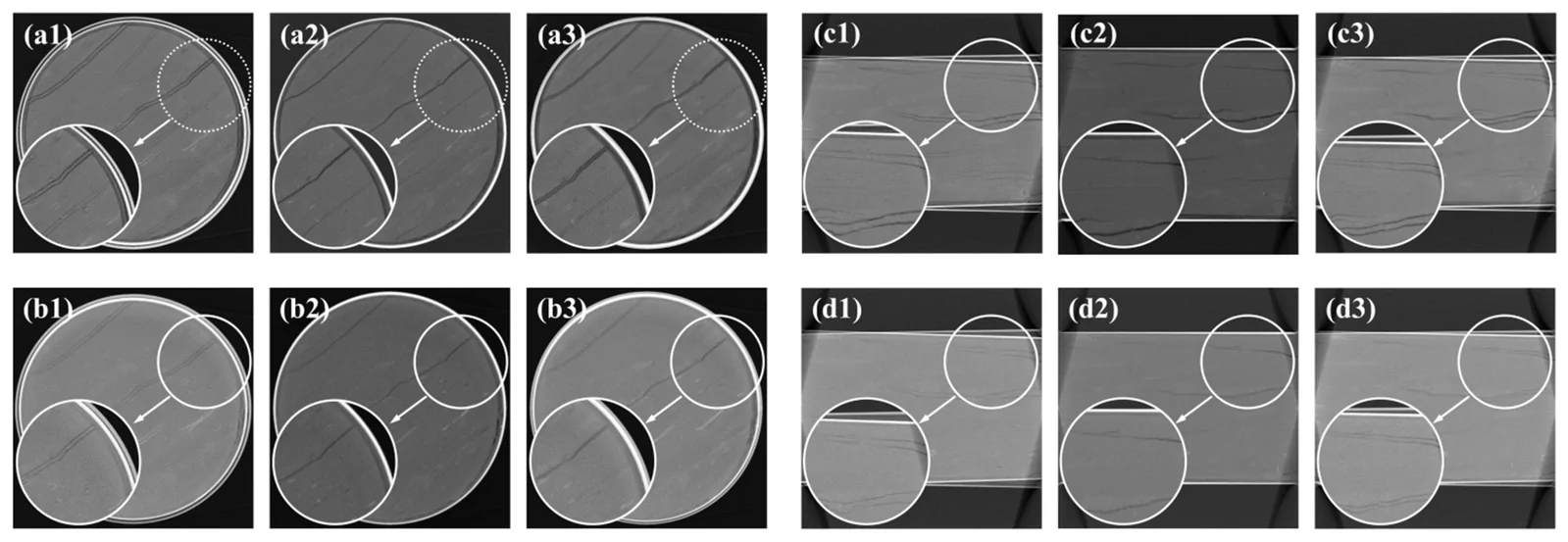
Reconstructed rock-core slices at the evaluated temperature stages: (**a1**) 100 °C transverse slice, uncalibrated; (**a2**) 100 °C transverse slice, projection-registration baseline; (**a3**) 100 °C transverse slice, proposed framework; (**b1**) 400 °C transverse slice, uncalibrated; (**b2**) 400 °C transverse slice, projection-registration baseline; (**b3**) 400 °C transverse slice, proposed framework; (**c1**) 100 °C longitudinal slice, uncalibrated; (**c2**) 100 °C longitudinal slice, projection-registration baseline; (**c3**) 100 °C longitudinal slice, proposed framework; (**d1**) 400 °C longitudinal slice, uncalibrated; (**d2**) 400 °C longitudinal slice, projection-registration baseline; and (**d3**) 400 °C longitudinal slice, proposed framework. Insets enlarge the indicated crack regions.

Inserting a calibration phantom during the thermal loading procedure was impractical. The evaluated projection registration baseline retained visible blur in several regions of the rock matrix (Figure 8).

The proposed refinement restricted detector-offset and orientation corrections to the physically feasible set and produced clearer crack boundaries in the displayed slices. Because independent time-resolved geometric ground truth was unavailable, this experiment demonstrates comparative improvement after estimating a scan-averaged effective correction—it does not demonstrate direct tracking of thermal drift. Any remaining intra-scan motion or slow geometric drift is expected to contribute to residual blur. A time-varying extension would require overlapping angular windows or a smooth temporal basis while preserving sufficient angular support.

### 3.4. Real-World Application III: Vertical CT of a Bluetooth Earphone

**Figure 9:**
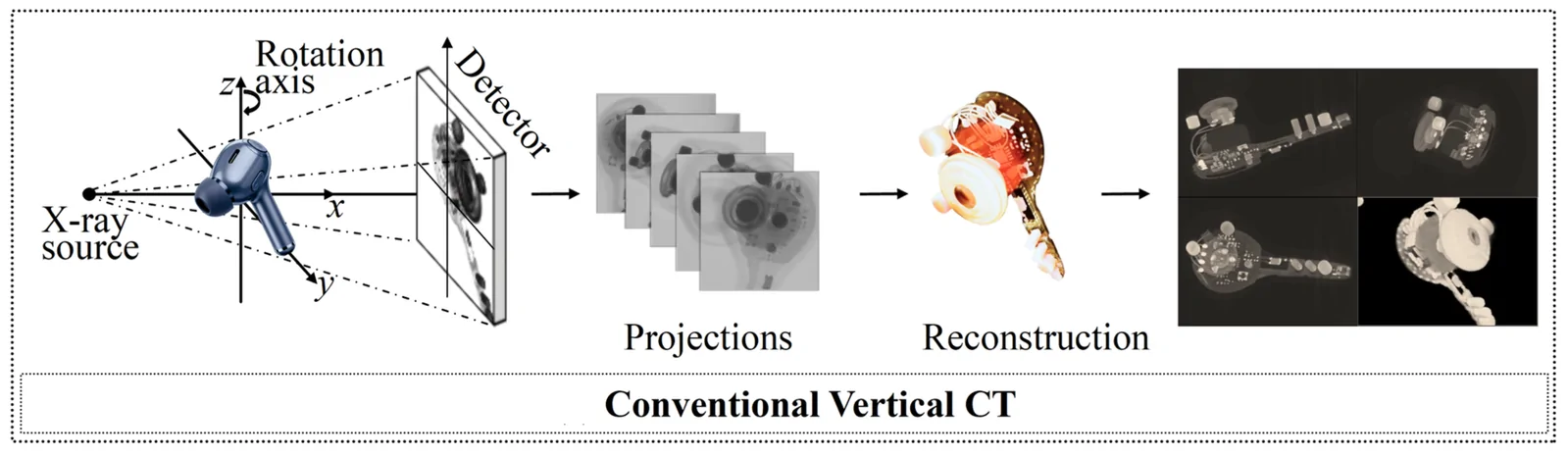
Vertical CT inspection of a commercial Bluetooth earphone containing high-density metal and fine microelectronic structures.

A commercial Bluetooth earphone was scanned using a Comet Yxlon FF35 vertical CT system to evaluate performance on a structurally complex object containing high-density metal components and fine microelectronic features (Figure 9). Metal-induced projection inconsistencies, continuous-spectrum beam hardening, sinogram processing, and edge ringing may coexist with geometric misalignment and can affect local edge-response measurements [6,7,8,30]. The proposed method addresses geometric reindexing only and is not a metal artifact reduction or image domain deconvolution algorithm.

**Figure 10:**
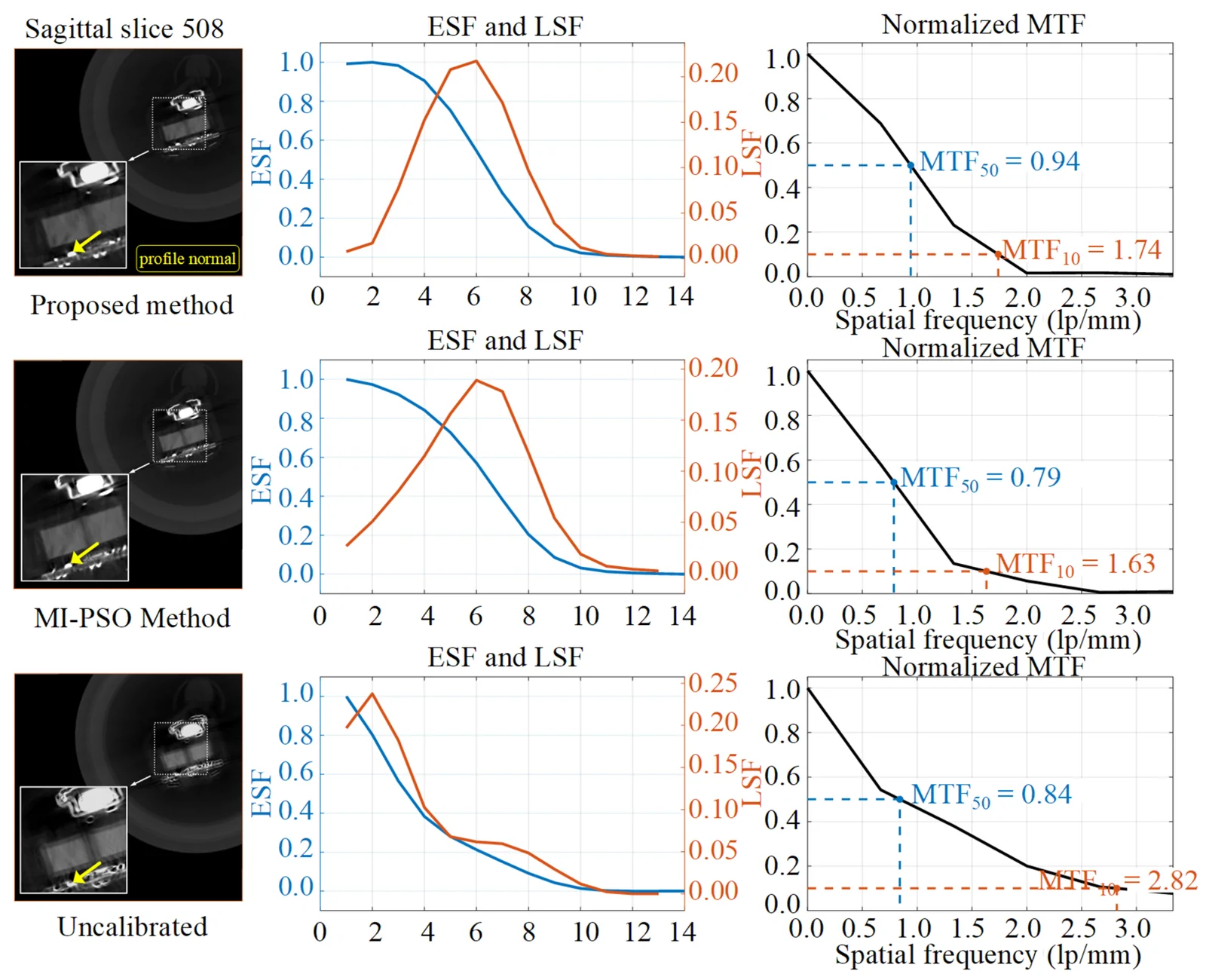
Sagittal slice comparison for the Bluetooth earphone. Rows show the proposed method, MI-PSO baseline, and uncalibrated state, while columns show the reconstructed slice, ESF/LSF profiles, and corresponding MTF curves. Dashed boxes indicate the sampled local region, and yellow arrows show the profile-normal direction used for ESF extraction.

**Figure 11:**
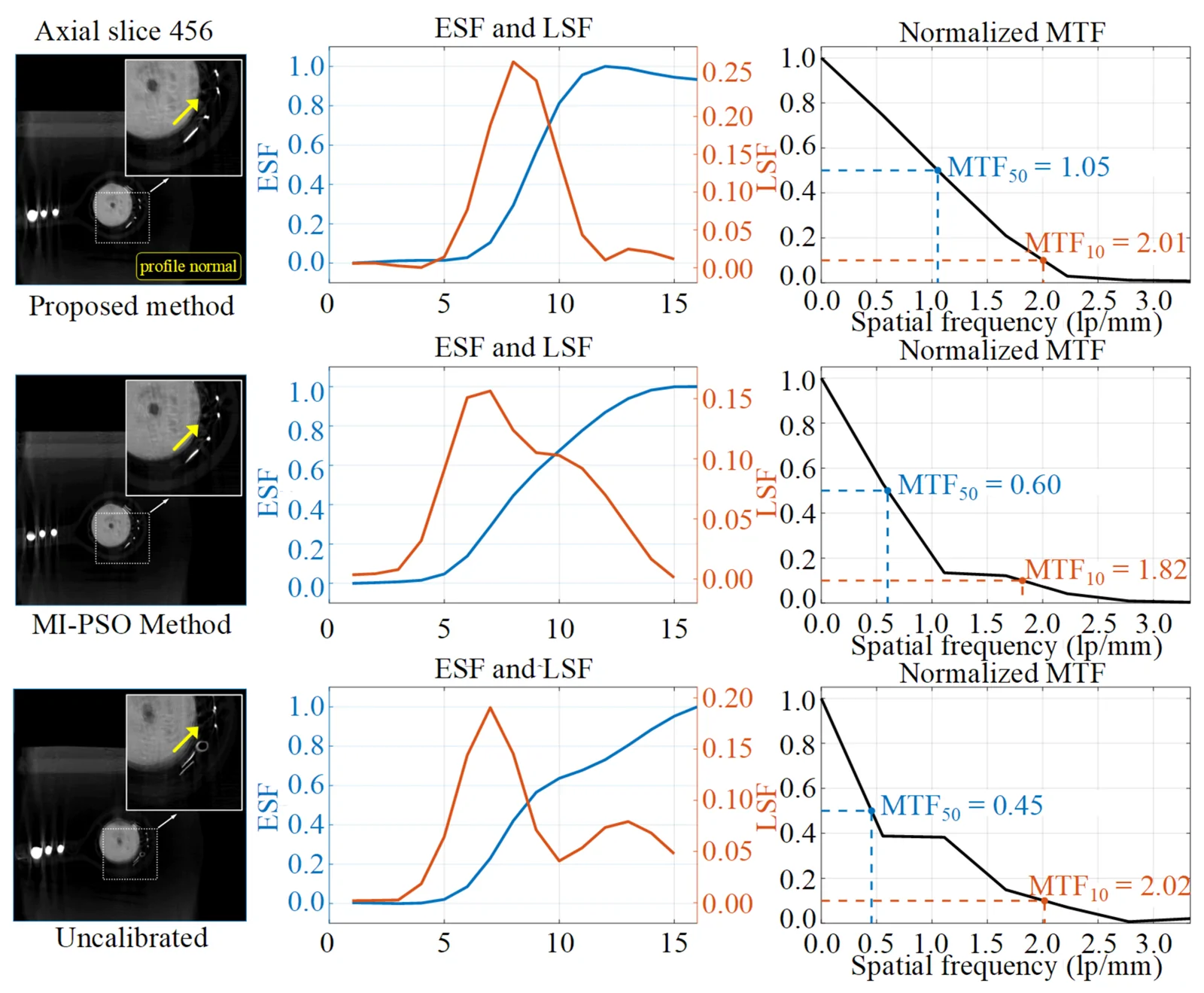
Axial slice edge-response comparison. Rows show the proposed method, MI-PSO baseline, and uncalibrated state. Dashed boxes indicate the sampled region, and yellow arrows show the profile-normal direction. The proposed reconstruction has a narrower central LSF peak and higher local MTF_50_, but its non-monotonic ESF makes the conventional 10–90% width unreliable.

The MI-PSO reconstruction retained radial streaks around several metal components in both the sagittal and axial slices (Figure 10 and Figure 11). The composite geometric objective reduced the influence of view-inconsistent streak components during the evaluated reconstruction. The resulting BGA package and microcircuit boundaries are sharper than in the corresponding MI-PSO reconstruction in the displayed regions. The volume was reconstructed using an isotropic voxel size of 0.15 mm.

For each evaluated slice, a local object boundary was selected, and the edge-spread function (ESF) was sampled along the profile-normal direction and averaged along the edge direction. Figure 10 and Figure 11 overlay the sampled region and a yellow arrow indicating the profile normal. The line-spread function (LSF) was obtained by differentiating the ESF, and the modulation transfer function (MTF) was calculated from the magnitude of the Fourier transform of the windowed LSF and normalized to its zero-frequency value. MTF_50_ and MTF_10_ denote the spatial frequencies at which the normalized MTF decreases to 0.5 and 0.1, respectively. These values are local, edge-response-derived estimates for relative comparison among reconstructions of the same dataset—they are not standardized measurements of the complete CT-system MTF. For a non-monotonic ESF, the conventional 10–90% threshold width is considered unreliable because multiple or unstable threshold crossings may occur.

MTF_10_ was interpreted together with MTF_50_, ESF behavior, and LSF FWHM because streak artifacts, noise, ringing, and non-monotonic edge responses can elevate the high-frequency tail without indicating improved boundary fidelity [6,7]. For example, the uncalibrated sagittal reconstruction has a higher MTF_10_ than the corrected reconstructions despite its substantially larger LSF FWHM. No single edge-derived metric is, therefore, used as conclusive evidence of geometric accuracy.

In the sagittal slice, the proposed method reduces the ESF 10–90% transition width to 4.52 pixels and the LSF full width at half maximum from 9.92 to 4.34 pixels. The sagittal MTF_50_ increases from 0.84 to 0.94 lp/mm, while the axial MTF_50_ increases from 0.45 to 1.05 lp/mm.

The axial slice results are metric-dependent. The proposed method reduces the LSF FWHM from 6.84 pixels for MI-PSO to 3.54 pixels and increases MTF_50_ from 0.60 to 1.05 lp/mm, indicating a narrower central LSF peak for the selected edge. However, the corresponding ESF is non-monotonic: it reaches a local maximum and then declines toward the material plateau. This overshoot/ringing behavior makes automated 10–90% threshold detection unstable, and the previous 7.40-pixel value is, therefore, not interpreted as a physical transition width and is marked as unreliable in Table 3. Because geometric correction, metal artifacts, noise, interpolation, and reconstruction processing were not independently decomposed, the observed overshoot is not attributed uniquely to the NNSS term. A controlled noise/artifact ablation is required for causal attribution.

### 3.5. Image-Quality Consistency Against a Phantom-Based Calibration Reference

**Figure 12:**
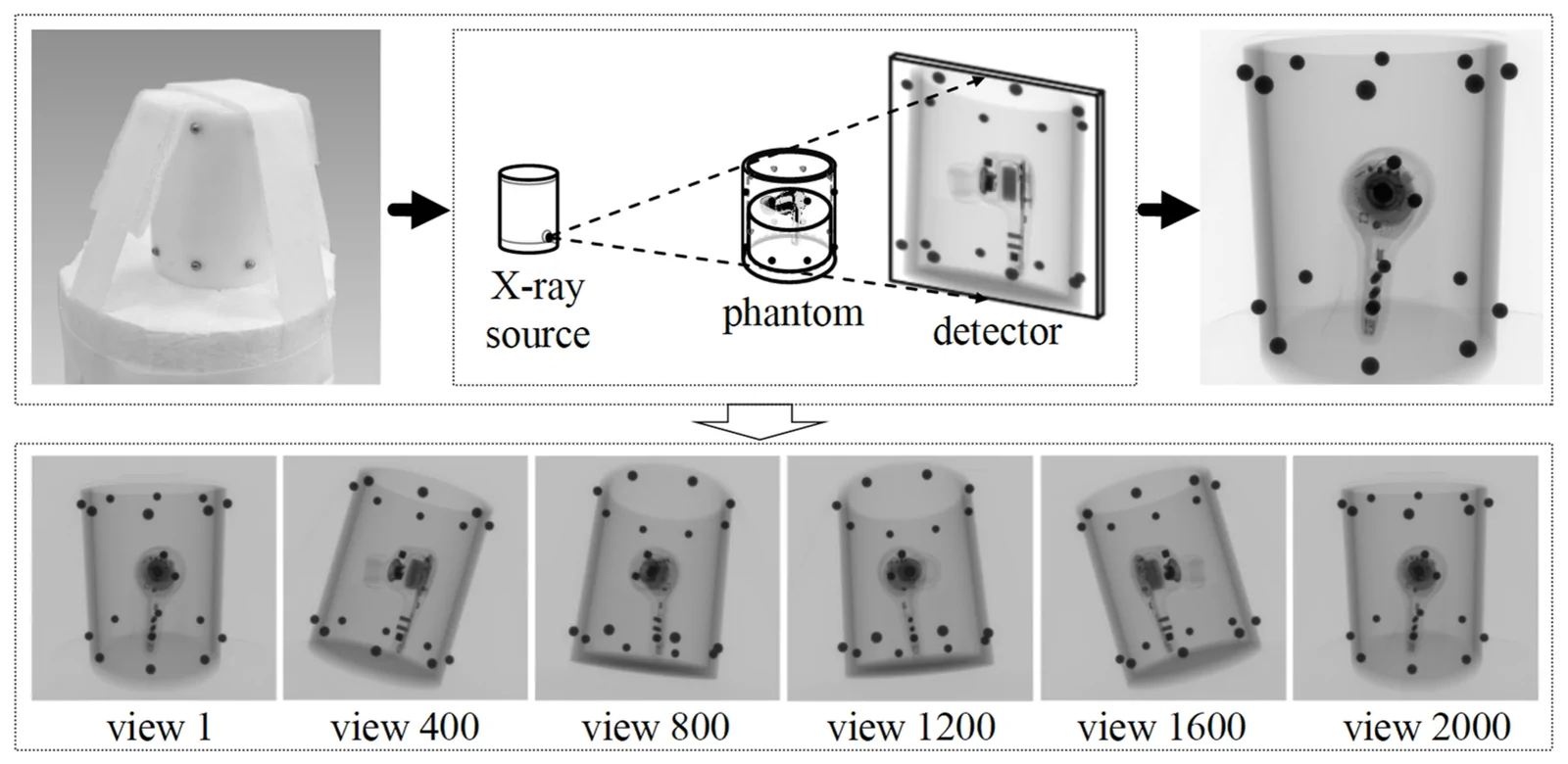
Metal ball-array phantom and projected marker features used to obtain the phantom-based reference geometry.

The proposed phantom-free reconstruction was compared with a reconstruction generated using geometry obtained from a metal ball-array phantom and an implementation of the Noo analytical method [31]. The phantom-derived geometry is used as a practical image-quality reference. Because the comparison is based on edge profiles and MTF curves rather than traceable dimensional measurements or direct parameter ground truth, it is not an independent validation of absolute dimensional accuracy. The phantom and representative projected marker features are shown in Figure 12.

**Figure 13:**
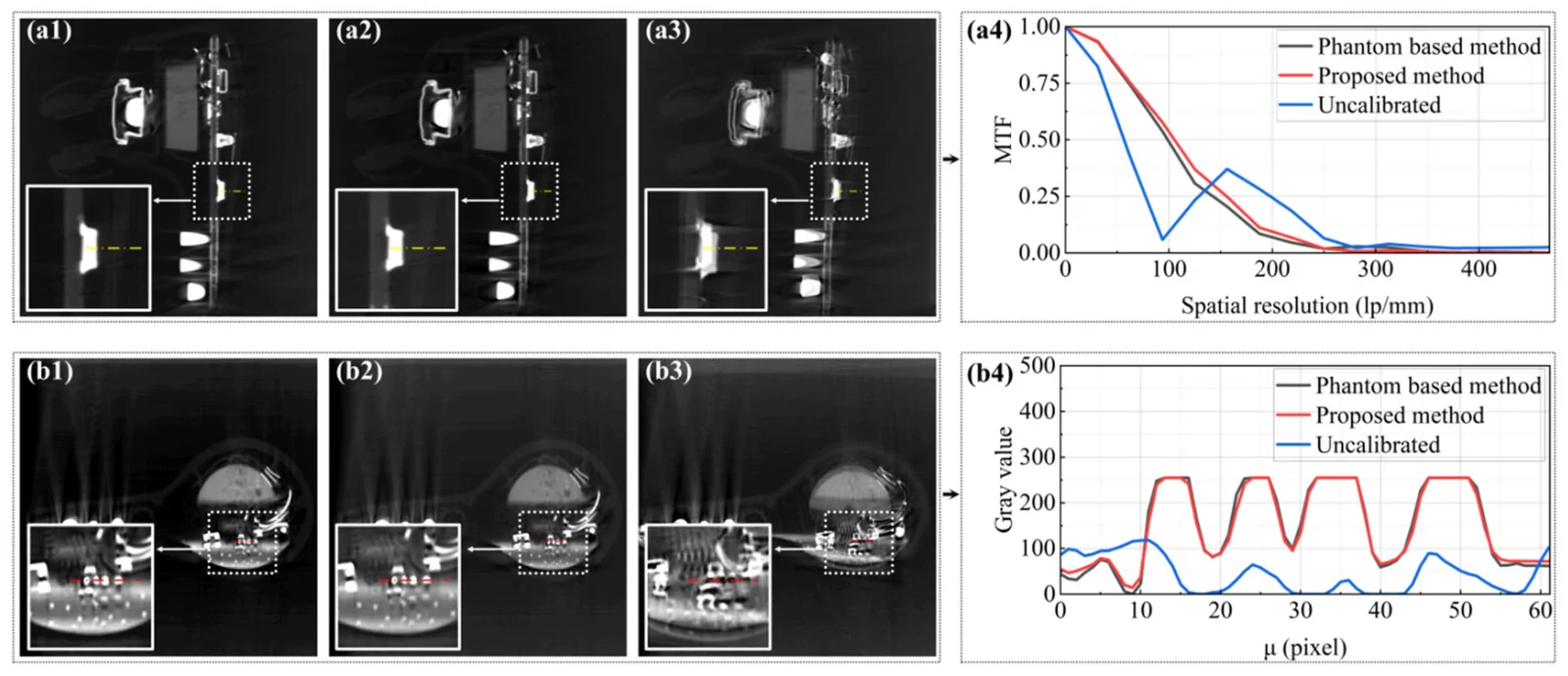
Image-quality comparison with the phantom-based reference calibration: (**a1**) sagittal reconstruction using the phantom-based method; (**a2**) sagittal reconstruction using the proposed method; (**a3**) uncalibrated sagittal reconstruction; (**a4**) MTF curves evaluated along the yellow dashed edge; (**b1**) axial reconstruction using the phantom-based method; (**b2**) axial reconstruction using the proposed method; (**b3**) uncalibrated axial reconstruction; and (**b4**) gray-value profiles sampled along the red dashed line. Insets enlarge the evaluated regions.

Figure 13 compares the uncalibrated, phantom-free, and phantom reference reconstructions. The gray-value profiles and MTF curves show that the phantom-free reconstruction approaches the edge-response characteristics of the phantom-based reference more closely than the uncalibrated result. This comparison demonstrates image-quality agreement for the selected feature but does not establish equivalence of the complete geometric parameter vector or absolute dimensional traceability.

The phantom-free refinement, therefore, provides a practical scan-specific alternative when placement of a calibration object during object acquisition is inconvenient. In this experiment, the local edge-response characteristics approach those of the phantom-based reference, but the comparison does not establish equivalence of the complete physical geometry.

### 3.6. Quantitative Comparison with a Public Projection-Consistency Baseline

**Figure 14:**
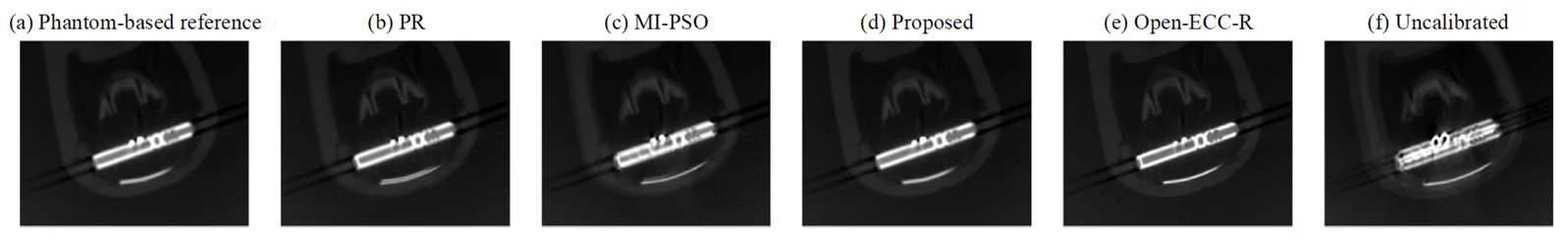
Same-location axial reconstruction comparison on the controlled industrial object reprojection benchmark: (**a**) phantom-based post hoc reference, (**b**) PR, (**c**) MI-PSO, (**d**) proposed framework, (**e**) Open-ECC-R, and (**f**) uncalibrated reconstruction. All panels use identical slice coordinates and display normalization.

To address the request for an experimentally evaluated projection-consistency comparator, Open-ECC-R, MI-PSO, PR, and the proposed framework were applied to the controlled reduced-resolution industrial object projection dataset described in Section 2.4.3. All methods used the same 72 views, 96 × 96 detector sampling, nominal geometry, reconstruction grid, axial slice range, and fixed evaluation region. Figure 14 shows representative reconstructions at the same axial location and with the same display normalization.

**Figure 15:**
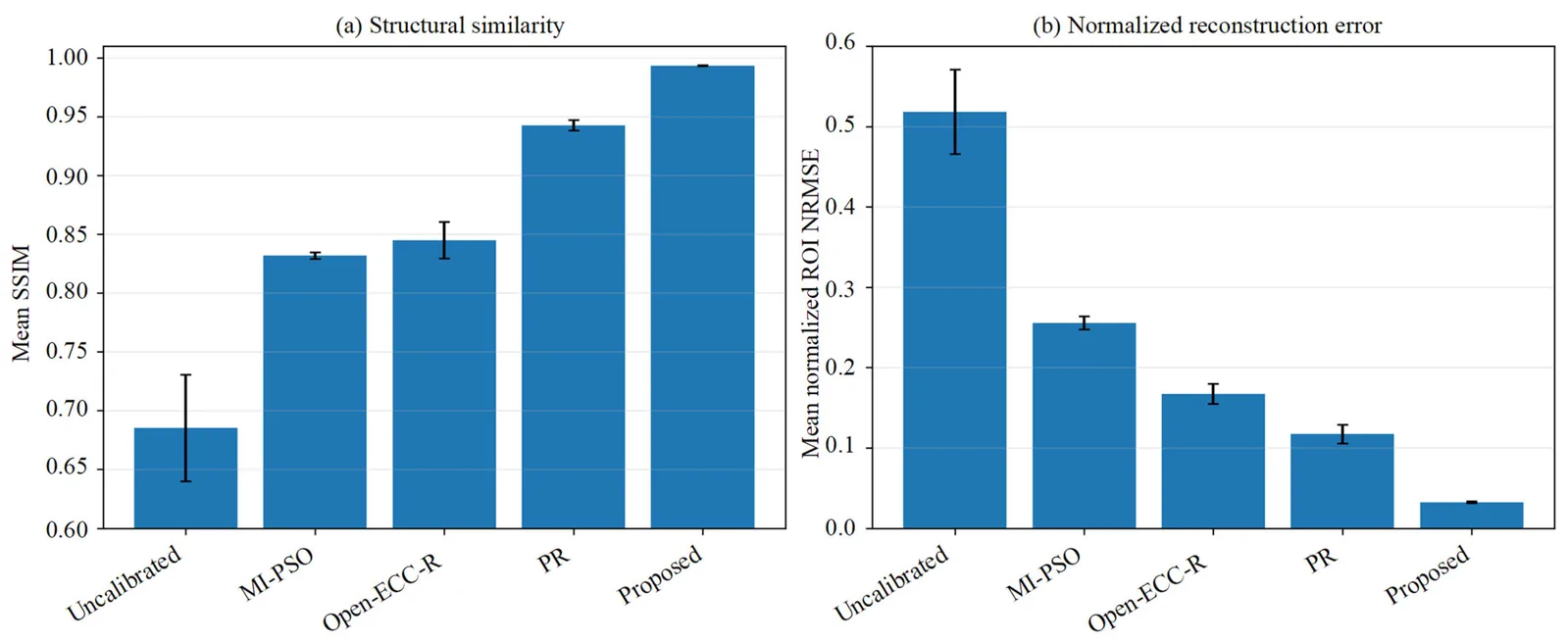
Quantitative comparison over 20 fixed-ROI axial slices: (**a**) mean SSIM and (**b**) mean normalized ROI NRMSE. Error bars denote the across-slice standard deviation.

Open-ECC-R provided a substantial improvement over the uncalibrated reconstruction: the mean SSIM increased from 0.6852 ± 0.0454 to 0.8450 ± 0.0155, the gradient-SSIM increased from 0.5111 ± 0.0246 to 0.6971 ± 0.0159, and the normalized ROI NRMSE decreased from 0.5180 ± 0.0526 to 0.1671 ± 0.0125. PR achieved a mean SSIM of 0.9425 ± 0.0044 and an NRMSE of 0.1172 ± 0.0117. The proposed method achieved the highest mean SSIM (0.9933 ± 0.0002) and gradient-SSIM (0.9773 ± 0.0015), together with the lowest NRMSE (0.0321 ± 0.0009) and the highest PSNR (45.052 ± 0.892 dB) among the evaluated phantom-free methods. Figure 15 and Table 4 summarize the across-slice quantitative results.

The ranking of the quantitative metrics agrees with the representative images: Open-ECC-R improves projection-consistency-based reconstruction quality relative to the uncalibrated state, while the proposed bounded CMT/NNSS refinement provides the closest agreement with the phantom-based reference under the evaluated conditions. Because the input projections were generated by controlled reprojection and the reference was used only after optimization, these results demonstrate comparative image-quality performance rather than raw scanner pose recovery or dimensional traceability.

## 4. Discussion

### 4.1. Mechanistic Interpretation of the Three-Stage Framework

The three stages address complementary numerical difficulties rather than establishing global or coordinate-wise identifiability. Stage I uses a fitted periodic CMT residual to reject trajectory-inconsistent coarse candidates. Stage II restricts translation- and rotation-dominant updates to a physically feasible box. Stage III evaluates the energy-normalized NNSS over fixed corrected rows and accepts a refinement only when the trajectory loss does not increase. The exact-ASTRA verification confirms the detector transformation, but the objective profile analysis shows that the surrogate minima need not coincide with the injected value for every coordinate. The method should, therefore, be interpreted as a bounded, reproducible local geometric refinement search whose output is validated through projection and reconstruction behavior.

### 4.2. Comparison with the Evaluated Baselines

MI-PSO was selected as a representative image similarity baseline and PR as a complementary correspondence-based baseline. Open-ECC-R was added in the present revision as a direct projection redundancy comparator based on the public epipolar-consistency formulation [22]. In the controlled industrial object benchmark, Open-ECC-R improved the mean SSIM from 0.6852 for the uncalibrated reconstruction to 0.8450 and reduced NRMSE from 0.5180 to 0.1671. PR obtained SSIM 0.9425 and NRMSE 0.1172, whereas the proposed framework obtained SSIM 0.9933 and NRMSE 0.0321. Thus, the projection-consistency baseline is effective relative to the uncalibrated state, but the proposed framework provides the closest agreement with the phantom-based post hoc reference among the methods evaluated under identical sampling and ROI conditions. These findings support comparative superiority for this benchmark—they do not establish universal superiority, absolute pose accuracy, or dimensional traceability.

### 4.3. Limitations and Future Work

Several limitations should be acknowledged. First, LCMT is a low-order screening surrogate and is invariant to ideal view-independent centroid shifts because the fitted basis contains a constant term, and it, therefore, cannot independently identify constant detector offsets. Second, NNSS is object-, trajectory-, cropping-, and preprocessing-dependent and does not independently identify every orientation coordinate. Third, the row-wise score does not explicitly model inter-row covariance, although full two-dimensional reindexing makes it indirectly responsive to vertical inconsistency. Fourth, the method searches bounded corrections around nominal *D*_SO_ and *D*_SD_ and does not recover unrestricted object scale or a complete seven-parameter geometry. Fifth, finite strict-decrease updates guarantee termination at a fixed resolution but not a global optimum. Sixth, one static equivalent correction is estimated for each scan, so residual thermal drift can remain as blur. Finally, the newly added Open-ECC-R comparison uses a controlled industrial object reprojection dataset rather than raw scanner projections; therefore, it strengthens the experimental comparison but is not a substitute for independent raw data or metrological validation. Future work will integrate directly identifiable projection-consistency information into the proposed hierarchy, investigate multi-row or low-rank-plus-sparse scores, model temporal drift, and validate the method using raw industrial projections with independent geometric references.

## 5. Conclusions

This study presents a three-stage phantom-free geometric refinement framework for bounded scan-specific correction around a nominal CBCT geometry. A fitted periodic CMT residual screens coarse candidates, physical bounds constrain block-coordinate updates, and an energy-normalized nuclear-norm score provides local projection correlation fine-tuning. The formulation explicitly treats these terms as surrogate objectives rather than as standalone proofs of five-coordinate physical identifiability.

The exact-ASTRA numerical check verified the detector axis convention and inverse reindexing operation, while three industrial applications showed clearer displayed boundaries and reduced visible blur relative to the evaluated baselines. The newly added controlled industrial object comparison directly evaluated MI-PSO, PR, Open-ECC-R, and the proposed method. Open-ECC-R increased mean SSIM from 0.6852 ± 0.0454 to 0.8450 ± 0.0155 and reduced NRMSE from 0.5180 ± 0.0526 to 0.1671 ± 0.0125 relative to the uncalibrated reconstruction. The proposed method achieved the highest mean SSIM of 0.9933 ± 0.0002 and the lowest NRMSE of 0.0321 ± 0.0009 among the evaluated phantom-free methods.

These results support the feasibility of scan-specific phantom-free geometric refinement when a calibration phantom is difficult to deploy during object acquisition. The scope remains limited to bounded effective corrections around nominal distances and to comparative projection/reconstruction evidence—the method does not establish unrestricted seven-parameter identifiability, coordinate-wise physical pose recovery, continuously time-varying calibration, global optimality, or absolute dimensional traceability.

## Figures and Tables

**Table 1:** Experimental datasets, scanner configurations, dominant geometric challenges, and evaluation evidence.

Dataset/Scenario	CT System	Pixel Pitch (mm)	Target Feature	Dominant Challenge	Evaluation Evidence
3.1. Numerical implementation check	Shepp–Logan/ASTRA	2.546/3.394	Detector reindexing	Axis convention and objective scope	NRMSE ratios, round-trip error, objective profiles
3.2. Horizontal gantry CT	Custom horizontal gantry	0.200	Tungsten particle boundaries	Gravity-dependent support deformation	Qualitative boundary comparison with MI-PSO
3.3. Temperature-stage in situ CT	Sanying nanoVoxel-4000	0.050	Rock-core cracks	Temperature-dependent scanner changes	Qualitative blur and crack boundary comparison with PR
3.4. Vertical micro-CT	Comet Yxlon FF35	0.139	Bluetooth earphone microelectronics	Metal streaks coupled with misalignment	Sagittal/axial MTF_50_: 0.94/1.05 lp/mm
3.6. Controlled industrial object reprojection benchmark	Comet Yxlon FF35/ASTRA	0.139	Bluetooth earphone ROI	Public projection-consistency comparison	SSIM, gradient-SSIM, NRMSE, PSNR

**Table 2:** Exact-ASTRA detector reindexing verification for the three injected perturbation levels.

Level	Injected Offsets (mm)	Injected Angles (°)	Corrected/Uncorrected NRMSE	Round-Trip NRMSE	Reduction
Small	[0.4, −0.3]	[0.35, −0.25, 0.40]	0.3523	0.0230	64.8%
Medium	[1.2, −1.0]	[1.10, −0.90, 1.30]	0.1365	0.0222	86.3%
Large	[2.5, −2.0]	[2.20, −1.80, 2.50]	0.0866	0.0233	91.3%

**Table 3:** Spatial resolution and edge-response metrics for the Bluetooth earphone dataset.

Method	Slice Orientation	MTF_50_ (lp/mm)	MTF_10_ (lp/mm)	ESF 10–90% Width, Pixels (mm)	LSF FWHM, Pixels (mm)
Uncalibrated	Sagittal Slice	0.84	2.82	5.04 (0.756)	9.92 (1.489)
	Axial Slice	0.45	2.02	8.14 (1.220)	6.41 (0.961)
MI-PSO Baseline	Sagittal Slice	0.79	1.63	4.79 (0.719)	4.77 (0.716)
	Axial Slice	0.60	1.82	6.86 (1.029)	6.84 (1.027)
Proposed Framework	Sagittal Slice	0.94	1.74	4.52 (0.678)	4.34 (0.652)
	Axial Slice	1.05	2.01	Unreliable †	3.54 (0.531)

† The proposed method axial ESF is non-monotonic and exhibits local overshoot followed by a decline. The automated 10–90% threshold width is, therefore, unstable and is not interpreted as a physical transition width. The reported LSF FWHM and MTF values should also be considered jointly with visible metal/streak artifacts.

**Table 4:** Quantitative comparison over 20 fixed-ROI axial slices using the phantom-calibrated reconstruction as a post hoc image-quality reference.

PSNR (dB)	NRMSE	Gradient-SSIM	SSIM	Method
20.945 ± 1.700	0.5180 ± 0.0526	0.5111 ± 0.0246	0.6852 ± 0.0454	Uncalibrated
27.037 ± 0.881	0.2556 ± 0.0081	0.6170 ± 0.0098	0.8318 ± 0.0028	MI-PSO
30.748 ± 0.478	0.1671 ± 0.0125	0.6971 ± 0.0159	0.8450 ± 0.0155	Open-ECC-R
33.846 ± 0.392	0.1172 ± 0.0117	0.8329 ± 0.0117	0.9425 ± 0.0044	PR
**45.052 ± 0.892**	**0.0321 ± 0.0009**	**0.9773 ± 0.0015**	**0.9933 ± 0.0002**	**Proposed**

Bold values indicate the best result for each metric.

## Data Availability

Data underlying the results presented in this paper are not publicly available at this time but are available from the corresponding author upon reasonable request.

## Supplementary Materials

The following supporting information can be downloaded at: https://www.mdpi.com/article/10.3390/s26165161/s1. Figure S1: Quantitative exact-ASTRA detector reindexing verification: (**a**) reduction of valid-mask projection NRMSE after inverse reindexing and (**b**) round-trip resampling NRMSE. Numerical values are listed in Table S1. Figure S2: One-dimensional projection NRMSE and CMT profiles using the automatically selected ASTRA/reindexing convention. Dashed lines indicate the injected coordinate values. Table S1: Exact-ASTRA detector reindexing verification for the three injected perturbation levels. Table S2: Coordinate profile minimum summary for the corrected ASTRA/reindexing convention. Table S3: Reduced-resolution computational benchmark—values are mean ± standard deviation over 60 diagnostic runs. Table S4: Component-level diagnostic under the fixed reduced-resolution configuration. Table S5: Parameter-setting sensitivity of the surrogate objectives. These values characterize objective behavior, not physical pose accuracy. Table S6: Initialization stability summary over 10 runs. Table S7: ±1% DSO/DSD model mismatch diagnostic. Objective values are reported to demonstrate model sensitivity, not distance parameter recovery.

## Abbreviations

The following abbreviations are used in this manuscript:

CBCT	Cone-beam computed tomography
CMT	Center-of-mass trajectory
ISOMAP	Isometric feature mapping
MI-PSO	Mutual-information particle swarm optimization
PR	Projection registration
MTF	Modulation transfer function
ESF	Edge-spread function
LSF	Line-spread function
FWHM	Full width at half maximum
BGA	Ball grid array
NNSS	Normalized nuclear-norm sinogram score
ECC	Epipolar-consistency condition
