# Supplementary material for: Phantom-Free Geometric Refinement for Industrial CBCT Using Physical Constraints and a Normalized Low-Rank Projection Prior
Source: Sensors (Basel). 2026 Aug 14;26(16):5161. doi: 10.3390/s26165161 (PMC13517192; doi:10.3390/s26165161)
Supplement: Supplementary file 1 [file sensors-26-05161-s001.zip › sensors-4470124-supplementary.pdf]

Supplementary Diagnostics for sensors-4470124

Phantom-Free Geometric Refinement for Industrial CBCT Using Physical Constraints and a Normalized Low-Rank Projection Prior

S1. Exact-ASTRA Detector-Reindexing Verification

A 1024^3 Shepp–Logan volume was downsampled to 128^3 voxels and forward projected using ASTRA Toolbox 2.0.1 with a 96 × 128 non-square detector and 72 views. The non-square detector was used to disambiguate the ASTRA output ordering from the physical detector u/v axes. The selected convention was permute [1 3 2], with array rows mapped to physical u, array columns mapped to physical v, positive u/v signs, and the physically expected inverse direction.

Table S1. Exact-ASTRA detector-reindexing verification for the three injected perturbation levels.

Level	Injected offsets (mm)	Injected angles (°)	Corrected/uncorrected NRMSE	Round-trip NRMSE	Reduction
Small	[0.4, −0.3]	[0.35, −0.25, 0.40]	0.3523	0.0230	64.8%
Medium	[1.2, −1.0]	[1.10, −0.90, 1.30]	0.1365	0.0222	86.3%
Large	[2.5, −2.0]	[2.20, −1.80, 2.50]	0.0866	0.0233	91.3%

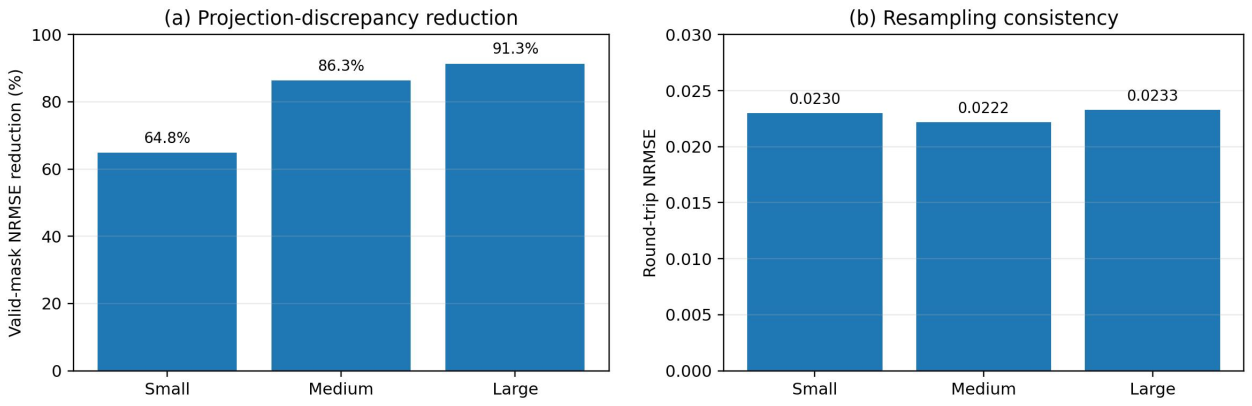

Figure S1. Quantitative exact-ASTRA detector-reindexing verification: (a) reduction of valid-mask projection NRMSE after inverse reindexing; (b) round-trip resampling NRMSE. Numerical values are listed in Table S1.

S2. One-Dimensional Objective Profiles

Table S2. Coordinate-profile minimum summary for the corrected ASTRA/reindexing convention.

Metric	Profiles with minimum at/near injected value	Interpretation

Metric	Profiles with minimum at/near injected value	Interpretation
Projection-domain NRMSE	15 of 15	Transformation/reindexing fidelity
Periodic CMT residual	0 of 15	Coarse object-dependent screening surrogate
NNSS	4 of 15	Local correlation surrogate; not stand-alone identification

Each correction coordinate was varied while the other four coordinates were fixed at their injected values. The orange curves show valid-mask projection NRMSE and the blue curves show the fitted periodic CMT residual. The projection NRMSE minima occur near the injected values after the detector convention is corrected, whereas the CMT minimum coincides with the injected value in 0 of 15 profiles. The NNSS minimum coincides with the injected value in 4 of 15 profiles. These diagnostics establish the scope of the surrogate objectives and motivate the removal of absolute five-coordinate parameter-recovery claims.

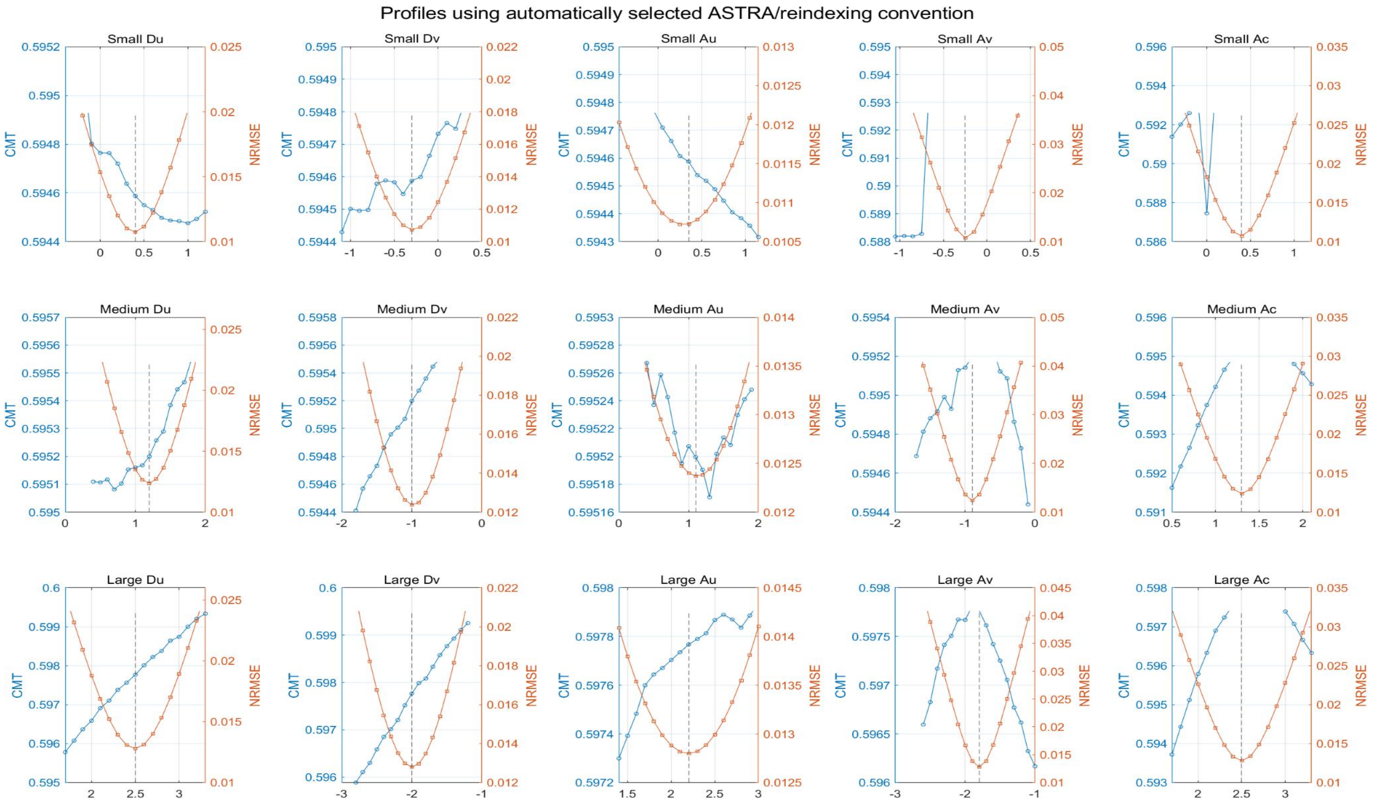

Figure S2. One-dimensional projection-NRMSE and CMT profiles using the automatically selected ASTRA/reindexing convention. Dashed lines indicate the injected coordinate values.

S3. Computational Benchmark

The benchmark was performed in MATLAB R2022a with ASTRA Toolbox 2.0.1 on an Intel Core i9-12900KF CPU, 128 GB DDR5 RAM, and an NVIDIA GeForce RTX 3090 Ti GPU. The diagnostic implementation used 36

views at 64 × 64 for coarse processing and 72 views at 96 × 96 for fine processing. Values in Table S3 are mean ± standard deviation over 60 repeated runs with fixed settings.

Table S3. Reduced-resolution computational benchmark; values are mean ± standard deviation over 60 diagnostic runs.

Quantity	Measured value	Interpretation
Stage I runtime	2.49±0.12 s	CMT candidate screening
Stage II runtime	2.89±0.12 s	Bounded block-coordinate refinement
Stage III runtime	5.67±2.32 s	NNSS local refinement
Total runtime	11.06±2.33 s	Excludes full-resolution reconstruction
CMT evaluations	204.7±7.2	Per diagnostic run
NNSS evaluations	22.3±13.0	Per diagnostic run

A standardized single-run benchmark required 10.31 s for the proposed implementation (229 objective evaluations), 6.51 s for the implemented MI-PSO benchmark (180 evaluations), and 2.88 s for the implemented projection-registration benchmark (71 evaluations). The two baseline benchmarks used reference-assisted objectives and are not accuracy-equivalent to the phantom-free surrogate objective; the values are provided only to indicate computational scale.

S4. Component and Parameter-Sensitivity Diagnostics

The following diagnostics characterize how the implemented hierarchy and predeclared settings affect surrogate values and runtime. Because CMT and NNSS are not coordinate-wise truth-identifying, the tables are not interpreted as physical-parameter accuracy or as a monotonic "more components is always better" claim.

Table S4. Component-level diagnostic under the fixed reduced-resolution configuration.

Variant	Final LCMT	Final NNSS	Runtime (s)	Interpretation
CMT screening only	0.01328	—	2.26	Coarse trajectory screening
+ block refinement (wide bounds)	0.00809	—	5.02	Lower surrogate value in a broader, mechanically weak feasible set
+ physical bounds	0.01272	—	5.09	Mechanically plausible bounded search
Complete framework (+ NNSS)	0.01289	1.57242	10.00	Local NNSS selection under the CMT constraint

Table S5. Parameter-setting sensitivity of the surrogate objectives. These values characterize objective behavior, not physical pose accuracy.

Factor	Value	Final LCMT	Final NNSS
Harmonic order	1	0.068925	1.398876

Factor	Value	Final LCMT	Final NNSS
Harmonic order	2	0.012899	1.576076
Harmonic order	3	0.011274	1.577867
NNSS ε	10^{-8}	0.012881	1.575652753
NNSS ε	10^{-6}	0.012881	1.575652685
NNSS ε	10^{-4}	0.012881	1.575645893
Bound multiplier	0.75	0.016226	1.538413
Bound multiplier	1.00	0.013093	1.576038
Bound multiplier	1.50	0.010143	1.643876

S5. Initialization and Fixed-Distance Model Diagnostics

Ten different initial correction vectors produced a narrow range of final surrogate values but different selected correction vectors, confirming initialization dependence. A separate exact-ASTRA diagnostic perturbed D_{SO} and D_{SD} by ±1% while keeping the five-coordinate refinement model fixed. The resulting changes are reported only as surrogate-objective sensitivity; they are not interpreted as recovery accuracy for D_{SO} or D_{SD}.

Table S6. Initialization-stability summary over 10 runs.

Quantity	Mean ± SD	Range
Final LCMT	0.013056 ± 0.000198	0.012737–0.013310
Final NNSS	1.576837 ± 0.004413	1.570454–1.582165
Runtime (s)	10.27 ± 1.86	7.93–14.08
Selected correction vector	Varied across runs	No initialization-independent physical solution claimed

Table S7. ±1% D_{SO}/D_{SD} model-mismatch diagnostic. Objective values are reported to demonstrate model sensitivity, not distance-parameter recovery.

D_{SO} error (%)	D_{SD} error (%)	Final LCMT	Final NNSS
0	0	0.357664	1.152567
−1	0	0.365226	1.149874
+1	0	0.330123	1.136853
0	−1	0.335800	1.134558

D_{SO} error (%)	D_{SD} error (%)	Final LCMT	Final NNSS
0	+1	0.344896	1.133112
−1	+1	0.329939	1.137026
+1	−1	0.348077	1.136669

S6. Applicability Boundaries

The diagnostics support bounded, scan-specific image-quality refinement around nominal geometry. They do not establish unrestricted D_{SO}/D_{SD} recovery, initialization-independent five-coordinate pose identification, severe sparse-view or limited-angle robustness, continuously time-varying calibration, or dimensional traceability. These conditions remain outside the validated scope and are stated explicitly in the revised manuscript.
